# Unfinished business: integrating individual decision-makers' experience and incentives to organizational performance feedback theory

**DOI:** 10.3389/fpsyg.2023.1166185

**Published:** 2023-07-05

**Authors:** Daniela Blettner, Serhan Kotiloglu, Thomas G. Lechler

**Affiliations:** ^1^Beedie School of Business, Simon Fraser University, Burnaby, BC, Canada; ^2^College of Business Administration, California State University San Marcos, San Marcos, CA, United States; ^3^School of Business, Stevens Institute of Technology, Hoboken, NJ, United States

**Keywords:** behavioral theory of the firm, Carnegie perspective, decision making, individual-level, meta-analysis, organizational-level, performance feedback

## Abstract

In this study, we analyze the role of individual decision-makers in organizational decision-making that is described by the Carnegie perspective. In particular, building on the Behavioral Theory of the Firm, we analyze the influence of decision-makers on organizational responses to performance feedback. Managers in organizations can influence the performance feedback process through their individual experiences. Moreover, they are motivated and controlled by incentives, which is another mechanism by which organizational decision-making can be influenced by individuals. While the Carnegie perspective acknowledges that decision-makers interpret performance feedback and initiate organizational responses, individuals are not as closely integrated to the organizational performance feedback process as some other—mostly organizational—conditions. Recently, several intriguing empirical studies have addressed the role of experience and incentives in the performance feedback process. However, their *cumulative* effect remained impossible to assess. We meta-analytically review 205 BTOF studies to test our hypotheses on the influence of decision-makers' experience and incentives on organizational responses to performance feedback. We show that decision-makers' job experience and domain expertise influence organizational responses to performance below aspirations, while incentives and compensation become relevant when performance is above aspirations. These results highlight the importance of individual decision-makers in explaining variations in organizational performance feedback decisions, offering exciting venues for psychology scholars to contribute to the Carnegie perspective.

## 1. Introduction

The study of Cyert, March, and Simon, the founders of the Carnegie perspective, sought to understand and theorize how individuals in organizations make decisions (Simon, 1947; March and Simon, 1958; Cyert and March, 1992)^1^. One of the core theories of this perspective, The Behavioral Theory of the Firm (BTOF) (Cyert and March, 1992), explains how organizational decision-makers interpret organizational performance feedback and respond with strategic actions. The theory predicts that organizations routinely engage in problemistic search (a search for solutions) if organizational performance is below their aspirations and that they stop searching if their performance is above aspirations. This has been shown to be the case for many diverse strategic actions (e.g., change, risk-taking, and innovation), but empirical results are inconsistent (Posen et al., 2018). For performance below aspirations, many studies find support for problemistic search (Greve, 2003), but others do not find such evidence (Audia and Greve, 2006). For performance above aspirations, some studies find a decrease in responses as a result of inertia, but others demonstrate an increase in responses (Kotiloglu et al., 2021). To refine the specificity of the initial theory, researchers have started investigating how individual differences of key decision-makers in organizations, such as their levels of narcissism, overconfidence, or power, impact their interpretation of performance feedback and strategic responses (Schumacher et al., 2020; Audia and Greve, 2021). This study contributes to this research stream by focusing on the role of individual decision-makers.

Managers in organizations can influence the performance feedback process in various ways, and one important way is through their individual experiences (Blagoeva et al., 2020; Gaba et al., 2022). Experience plays a central role in both learning from feedback and decision-making, which are central themes of the Carnegie perspective (Cyert and March, 1992; March, 2008, 2010). Experience can take many forms. Performance feedback is a form of experience (Cyert and March, 1992). Learning curves is a form of experience that stems from the history of costs and efficiencies (Argote, 1999). The outcomes experienced by similar others are another form of experience (Cyert and March, 1992). Nonetheless, there is still much that we do not know about the influence of decision-makers' experience. In this study, we distinguish between job experience (i.e., knowledge gained on the job as CEO/key decision-maker through trial-and-error learning) and domain expertise (i.e., knowledge gained during their education, training, and prior functional experience). While domain expertise is based on norms and knowledge generated by society, an individual's job experience is more open to their cognitive biases as it is dependent on personal experiences. We argue that decision-maker job experience and decision-maker domain expertise differ in their effect on how decision-makers interpret feedback information and the range of strategic actions that they consider.

In the process of making decisions about strategic actions in response to performance feedback, decision-makers are motivated and controlled by incentives. While the impact of incentives on the individual decision-maker is conceptualized by the BTOF, it has only recently found its way into the empirical literature (Harris and Bromiley, 2007; Lim and McCann, 2014). In our study, we differentiate performance-based incentives (such as options and bonuses) from compensation (such as salary). Performance-based incentives address the agency problem and are designed to motivate decision-makers to increase risk-taking on behalf of the organization (Wiseman and Gomez-Mejia, 1998). We argue that performance-based incentives and compensation motivate risk-taking and strategic action, proposing stronger responses for performance-based incentives, but that performance-based incentives and compensation inhibit risk-taking above aspirations.

Recently, a sufficient number of studies that include experience and incentives in performance feedback have been published, enabling us to use meta-analytic methods to test their influences on organizational responses to performance feedback. Meta-analytic methods allow us to assess the overall cumulative effect of these individual-level factors on organizational responses, which is not possible with other research designs that are constrained with specific individual-level variables and organizational responses studied. In this study, we draw on cumulative empirical evidence from 205 BTOF studies to systematically analyze the effect of the individual decision-maker on organizational responses (Aguinis et al., 2011). In our analyses, we include studies that analyzed many diverse strategic actions, including organizational search, risk-taking, strategic change, and R&D intensity.

Our study calls attention to empirical patterns that can be drawn from accumulated evidence of four decision-maker centric variables that are highly relevant to organizations.

## 2. Theoretical context

### 2.1. Organizational responses to performance feedback in the BTOF

Understanding how decision-makers in firms respond to performance feedback is one of the core concerns of the Carnegie perspective (Gavetti et al., 2007). To explain the process, the BTOF draws on the concept of bounded rationality. Building on this premise, performance feedback research within the BTOF proposes that firms respond differently to performance below and above aspirations (Greve, 2003). Empirical evidence generally supports that firms engage in problemistic search for solutions to their performance shortfalls, resulting in increased responses (Greve, 2003), but some empirical studies report reduced search (Audia and Greve, 2006). Audia and Greve (2021) suggest two accounts for variations in responsiveness to low performance: either organizations switch their attention from the aspiration level to the survival point; or they assess low performance in a self-enhancing way (Audia and Brion, 2007; Jordan and Audia, 2012). Such a self-serving interpretation of feedback reduces the need to act in response to performance feedback below aspirations (Audia and Brion, 2007; Jordan and Audia, 2012).

For responses above aspirations, the BTOF predicts that organizations do not search or engage in strategic actions, relying instead on the exploitation of their competencies (Levinthal and March, 1993). A firm's aspiration level is a highly salient marker differentiating success from failure. When the risk of falling below the aspiration level is higher than the perceived gain from performance above the aspiration level, firms tend to become inert or complacent. In line with this argumentation, many empirical studies demonstrate that firms decrease their responses if they reach or exceed their aspiration level: for instance, organizations are less likely to change (Greve, 1998) or launch fewer products (Greve, 2003). Audia and Greve (2006) also found that firms are relatively insensitive to performance above the aspiration level and attribute this to inertia (particularly with large firms). However, empirical results are also controversial (e.g., Shinkle, 2012): Firms may also increase risk-taking (e.g., Singh, 1986), innovation (e.g., Nohria and Gulati, 1996; Chen and Miller, 2007), and change (e.g., Kraatz and Zajac, 2001) responses.

Overall, the empirical discourse on organizational responses to performance above aspirations is convoluted: organizations may increase or decrease responses to performance above aspirations depending on several contingencies (Blettner et al., 2019). Some researchers have addressed these variations in responses to performance above aspirations from an organizational perspective and had identified firm size and slack (Singh, 1986; Greve, 2003) as well as variations in past performance (Ref and Shapira, 2017) as possible contingencies that influence responses to performance above aspirations. However, while the Carnegie perspective acknowledges that such decisions are initiated by the key decision-makers in organizations (Cyert and March, 1992), the role of individuals in organizational decision-making processes is not fully integrated in the theory. Like other researchers, who have recently begun to address this lack of integration, we are interested in analyzing the role of individuals in this process.

### 2.2. Prospect theory from the Carnegie perspective

Boundedly rational behavior is assumed in the Carnegie perspective when theorizing that decision-makers become loss averse when they perform below aspirations (Gavetti et al., 2007). This theorizing is in line with prospect theory which predicts loss aversion for performance below aspirations and risk aversion for performance above aspirations, with the value function being concave for gains and convex for losses and about twice as steep for losses as for gains (Kahneman and Tversky, 1979; Greve, 2003; Kahneman, 2011). In both theories, comparisons with a reference point influence behavior. It is important to note the Carnegie perspective allows for variation in responsiveness while prospect theory assumes a choice between two invariant alternatives. Kahneman (2011) summarizes this research by saying that the “great majority of people” are risk averse and “most people” are loss averse (Kahneman, 2011: 280). Kahneman and Tversky (1979)'s original experiments on prospect theory show that between 58% and 92% of subjects are prone to prospect theory. Gächter et al. (2022) show that “71% of people displayed loss aversion in risky choice.” Nearly 79% show this decision behavior repeatedly, from one decision to the next (Glockner and Pachur, 2012). Accordingly, we can assume that prospect theory is widespread decision-making behavior. However, decision behavior is likely also influenced by individual differences. Such differences can be demographic, for instance, the decision-maker's age, gender, education, disposition, affect, mood, or information processing (Trepel et al., 2005; Pachur et al., 2008, 2017; Hönl et al., 2017; Gächter et al., 2022). One of the differences between the Carnegie perspective and prospect theory is that the Carnegie perspective is an organizational-level theory that considers experience and incentives of the individuals within the organization while prospect theory is a theory of individual choice.

Risk-taking plays a role in decisions in response to organizational feedback. Scholars have proposed and found evidence that individual differences among decision-makers—such as their self-efficacy (Audia et al., 2000), power (Blagoeva et al., 2020; Audia and Greve, 2021), narcissism (Chatterjee and Hambrick, 2011; Jordan and Audia, 2012; Steinberg et al., 2022), regulatory focus (Ahn et al., 2020), and overconfidence (Schumacher et al., 2020)—influence how they interpret organizational performance feedback and how much risk they take in response to this feedback. Individual personal factors (such as overconfidence, hubris, and narcissism) can motivate decision-makers to self-enhance; this means they increase the positivity of their self-views to protect themselves from negative feedback—which leads to less risk-taking and responsiveness to performance feedback (Sedikides and Strube, 1997; Audia and Brion, 2007; Jordan and Audia, 2012). Decision-makers' experience also influences the processing of feedback information (Blagoeva et al., 2020; Gaba et al., 2022). Blagoeva et al. (2020) suggested that experience reduces the need to self-enhance. However, their empirical evidence does not support this reasoning.

Decision-makers' motivation to respond to performance feedback is also likely affected by external conditions such as the incentives intended to reward effort, risk-taking, and attained performance. In experiments, Etchart-Vincent and l'Haridon (2010) tested three monetary incentive schemes and found differences among the incentive schemes for the gain domain but not for the loss domain. Gächter et al. (2022) showed that loss aversion increases with income and wealth. In the organizational context, incentives can also influence the weighing of risk and thus affect responses to performance feedback (Harris and Bromiley, 2007; Lim and McCann, 2014).

## 3. Hypothesis development

A central theme in the Carnegie perspective is experience (Levitt and March, 1988; Cyert and March, 1992). March (2008: 90) proposes that decision-makers gain in two important ways from experience: they gain knowledge about the world and confidence in their experiential knowledge. The argumentation in recent empirical BTOF studies on the role of experience with respect to responses to performance feedback has centered on the (over)confidence decision-makers gain from experience. Gaba et al. (2022) have shown that prior career experience impedes decision-makers' ability to recognize and respond to performance feedback below aspirations. They attribute this process to overconfidence, i.e., an overestimation of knowledge that decision-makers gain from experience. In contrast, Blagoeva et al. (2020) hypothesized that, when performance falls below aspirations, decision-makers become more confident and more responsive to performance feedback. However, their results showed the opposite: Experience was associated with reduced responses.

### 3.1. Decision-makers' job experience and domain expertise

In this study, we differentiate between decision-maker job experience and decision-maker domain expertise because they differ in their effect on the interpretation of performance feedback. We see differences in the (over)confidence mechanism, arguing that job experience is associated with overconfidence, while domain expertise is related to confidence. Importantly, we also see differences between job experience and domain expertise in terms of what knowledge about the world decision-makers gain, arguing that job experience is narrow and highly sensitive to biases while domain expertise allows for broader and less biased knowledge. For job experience and domain expertise, we develop hypotheses only for performance below aspirations. While we do not have theoretical predictions on the influences of job experience and domain expertise for performance above aspirations, we explore these relationships empirically and report the results.

#### 3.1.1. Decision-makers' job experience

As decision-makers (e.g., CEOs) constantly face novel and uncertain situations, they need to rely on trial-and-error or experiential learning. In this situation, “learners are dealing with small samples of poorly designed experiments” (March, 2008, p. 89). When learning experientially from small samples, decision-makers are prone to biases, for example, sampling bias since they tend to extrapolate from a very small sample of experiences during their tenure as CEO and under-sample rare events (March, 1991; Fox and Hadar, 2006). They also suffer from status quo bias due to their strong belief in the current strategy (Finkelstein and Hambrick, 1990; Wangrow et al., 2019). Over time, decision-makers tend to become myopic, focusing on successful actions and sampling these again (Levinthal and March, 1993). As their information processing is restricted by limited possibilities (Miller, 1991), decision-makers develop a particular repertoire of responses (Finkelstein and Hambrick, 1990). The rules that decision-makers derive through learning from experience are embedded in the context from which they originate. This makes them less sensitive to situation-specific factors (Ert, 2012). Their information processing is biased, further leading to biased analyses of the situation they are facing. In short, experiential learning limits the quality (e.g., reliability and validity) of the knowledge gained.

The more experienced decision-makers are in their job, and the more they become identified and enmeshed with previous decisions, the more they are subject to attribution bias (Alicke and Sedikides, 2009), especially when confronting low performance (Gaba et al., 2022). For this reason, we expect that decision-makers become less responsive to performance below aspirations as their job experience increases. Having a deeper pool of experiences means having a variety of successful experiences in the past (Gaba et al., 2022), which decreases the urgency of reacting to a recent performance shortfall and facilitates inaction.

Most decision-makers are not fully aware of the extent to which the knowledge base they acquired through experiential learning is biased and thus overestimate the amount and quality of knowledge that they gained, despite the biases that undermine it. Because their faith in their knowledge base and prior actions is high, they become overconfident and less responsive to performance feedback. Thus, we expect a weaker increase in responses to performance below aspirations when job experience is considered. We propose the following hypothesis:

***Hypothesis 1:*** *Studies that include decision-makers' job experience show a weaker increase in responses to performance below aspirations than those that do not include job experience*.

#### 3.1.2. Decision-makers' domain expertise

Decision-makers also have domain expertise, consisting of the knowledge and skills that they acquired in a particular knowledge domain, either accumulated through functional expertise or educational expertise. This knowledge is built on a specific discipline, and it is based on the norms of the profession or educational background. The rules that constitute this knowledge body are dissociated from the context in which they were created (March, 2008). Therefore, this body of knowledge is more generalizable, and it provides a more comprehensive understanding of situations, strategic options in response to feedback, and their potential consequences. This enables decision-makers to gain a more comprehensive understanding, generating multiple strategic options in response to feedback. Domain expertise gives them access to a wider range of beliefs. Their knowledge is broader, and this is reflected in a broader set of options for strategic responses to performance feedback.

As they gain more competence through accumulating domain expertise, they become increasingly confident in their abilities. As opposed to on-the-job experiential learning, domain expertise is accumulated in more diverse, educational, or professional situations. As such, it is more generalizable and less biased. Decision-makers can more accurately assess their domain expertise, and greater domain expertise leads to confidence and a readiness to act in response to feedback. They may also be less threatened by performance below aspirations because of their domain expertise, and they also ensure they have a wide range of alternative employment options. Their increased confidence and greater alternative options allow decision-makers with greater domain expertise to act more readily on performance than those with less domain expertise. As a result, we expect a stronger increase in responses to performance below aspirations when domain expertise is considered and propose the following hypothesis:

***Hypothesis 2:*** *Studies that include decision-makers' domain expertise show a stronger increase in responses to performance below aspirations than those that do not include domain expertise*.

### 3.2. Incentives as motivations for decision-makers

In their theorizing on organizational decision-making, Carnegie scholars have identified incentives as an important means to influence decision-making and align the interest of the individual decision-maker with the interest of the organization (March and Simon, 1958; Cyert and March, 1992). While initially March and Simon (1958) discuss how incentives can motivate employees, they later focus their discussion of incentives on key decision-makers, arguing that incentives are key to managers' learning since they motivate them to accept information and change their behavior (Cyert and March, 1992).

Detailed discussions about the effects of rewards on the responses of individuals to feedback can be found in the psychology literature (Kluger and DeNisi, 1996). Here, incentives or rewards are understood as motivating core behavioral principles for human responses to feedback. In this literature, the research on executive compensation (Gomez-Mejia and Wiseman, 1997; Devers et al., 2016) represents the most relevant literature to our argument because it specifically addresses how organizational decision-makers respond to rewards or incentives.

One stream within the larger literature on executive compensation presents a behavioral, bounded rationality perspective on executive compensation, the Behavioral Agency Model (BAM) (Wiseman and Gomez-Mejia, 1998). BAM takes the decision-makers' personal wealth and corresponding aspirations as reference points and therefore is very close to March's original models (March and Shapira, 1992). BAM centers on risk bearing (i.e., the extent to which executives are likely to perceive risk to their personal wealth) and predicts that executives will react conservatively to organizational performance above aspirations because they expect a gain in wealth (Wiseman and Gomez-Mejia, 1998) and any additional risk-taking on behalf of the organization might jeopardize this personal gain. When firm performance is below aspirations, decision-makers anticipate loss to personal wealth and, due to loss aversion, are willing to engage in greater organizational risk-taking. Therefore, the predictions of BAM match those of the Carnegie perspective (March and Shapira, 1992). However, the mechanism outlined by Wiseman and Gomez-Mejia is tied to anticipated personal rather than organizational gain or loss, as in the Carnegie perspective. This is in line with prospect theory, which assumes loss aversion of the individual. Accordingly, in the BAM model, the motivation for behavior originates in individual rather than organizational concerns. Integrating the insights from BAM into the theoretical framework of the Carnegie perspective allows us to explain how differences among key decision-makers' incentives impact organizational responses to performance feedback.

In this study, we differentiate decision-makers' performance-based incentives (e.g., bonus and options) from their compensation (e.g., salary). While we argue that both incentives and compensation affect responses to performance feedback, we propose that the influence of performance-based incentives is stronger than the influence of compensation.

#### 3.2.1. Performance-based incentives

Performance-based incentives change frequently, often yearly, and decision-makers thus tend to be sensitive to organizational performance. When their firms perform below aspirations, decision-makers who receive greater performance-based incentives have more of their personal wealth at risk than those receiving fewer: They face a greater cost of failure. For instance, if they hold stock options, they face greater risk of losing those options. Since individuals who receive greater incentives anticipate greater losses, they become more loss averse and are willing to take more risks to avoid the anticipated loss (Wiseman and Gomez-Mejia, 1998). As such, we expect a stronger increase in responses to performance below aspirations when performance-based incentives are considered and propose the following hypothesis:

***Hypothesis 3a:*** *Studies that include decision-makers' performance-based incentives show a stronger increase in responses to performance below aspirations than those that do not include performance-based incentives*.

Our prediction for responses to performance above aspirations is different. Here, those individuals who receive high incentives experience greater risk, potentially losing more. Therefore, these individuals will become risk averse. Our reasoning builds on Lim and McCann (2014), who showed that CEOs with higher variable pay, in the form of stock options, tend to be loss averse and are conservative when organizational feedback exceeds aspirations. Therefore, we expect a weaker increase in responses to performance above aspirations, as well as a weaker decrease in responses to performance above aspirations when performance-based incentives are considered. Thus, we propose the following hypotheses:

***Hypothesis 3b:*** *Studies that include decision-makers' performance-based incentives show a weaker increase in responses to performance above aspirations than those that do not include performance-based incentives*.
***Hypothesis 3c:*** *Studies that include decision-makers' performance-based incentives show a weaker decrease in responses to performance above aspirations than those that do not include performance-based incentives*.

#### 3.2.2. Compensation

Decision-makers receive compensation, for instance, their salaries, regularly. Compensation differs from performance-based incentives because it is long-term and more stable. Decision-makers rely on their salary for recurring expenses and consider it an endowment (Larraza-Kintana et al., 2007). The higher their compensation is, the more loss averse decision-makers become with regard to this endowment. In an attempt to protect future compensation, decision-makers take fewer strategic risks and reduce their responses to performance feedback (Wiseman and Gomez-Mejia, 1998). As such, we expect a stronger increase in responses to performance below aspirations when compensation is considered and propose the following hypothesis:

***Hypothesis 4a:*** *Studies that include decision-makers' compensation show a stronger increase in responses to performance below aspirations than those that do not include compensation*.

When considering their future risk-taking in response to feedback above aspirations, decision-makers experience instant endowment of their anticipated compensation and tend to become loss averse (Wiseman and Gomez-Mejia, 1998). To protect the anticipated compensation, which in their mental accounting already belongs to them, they will be inclined to take risks to stay above aspirations. Therefore, we expect that decision-makers with higher compensation are less prone to complacency when their firms perform above aspirations than those who receive lower compensation. However, highly reliable compensation does not motivate excessive risk-taking above aspirations. Thus, we expect a weaker increase in responses to performance above aspirations, as well as a weaker decrease in responses to performance above aspirations when compensation is considered. Accordingly, the hypotheses are as follows:

***Hypothesis 4b:*** *Studies that include decision-makers' compensation show a weaker increase in responses to performance above aspirations than those that do not include compensation*.
***Hypothesis 4c:*** *Studies that include decision-makers' compensation show a weaker decrease in responses to performance above aspirations than those that do not include compensation*.

## 4. Methods

To test our hypotheses, we followed recent meta-analyses in the organizational performance feedback and strategic management literature (Crook et al., 2008; Vanneste et al., 2014; Bilgili et al., 2016; D'Oria et al., 2021; Kotiloglu et al., 2021, 2023; Blettner et al., 2023) to compare the effects of performance feedback models that included and excluded decision-makers' job experience, domain expertise, performance-based incentives, and compensation in their analyses.

### 4.1. Sample

To identify and select appropriate studies for inclusion in our analysis, we searched for studies that analyze the effects of organizational performance feedback. Our sample selection criteria and process are summarized in Table 1. Our final sampling resulted in 205 empirical studies with 516 effect sizes and a total of 3,386,451 firm-year observations. Following Aguinis et al. (2018) and Combs et al. (2018), we report sample size, sample characteristics (i.e., time and location of data collection), and coding information for each study^2^.

**Table 1:** Sample selection and criteria.

**Step**	**Procedure**	**Number of studies**	**Notes**
1: Initial literature search	Using the following keywords for our searches of all journals included in the ABI/INFORMS and Web of Science databases: “aspiration level”, “attainment discrepancy”, behavioral theory of the firm”, “organizational change”, “organizational decision-making”, “organizational search”, “performance feedback”, “problemistic search”, “risk-taking”, “slack search”, and combinations of these terms.	263 new studies, added	- The resulting studies were published between 1987 and 2021. While we did not expect to find any studies on organizational performance feedback theory before the publication of A Behavioral Theory of the Firm (Cyert and March, 1963), the start for our analysis, 1987, emerged from our search. - All the studies in our sample reported at least one effect size for performance feedback and organizational responses.
2: Backward search	Through the references of the identified studies	Seven new studies, added	
3: Identify unpublished studies	Solicited our request for unpublished studies through Academy of Management (AoM) lusters. We published our request for unpublished studies in several divisions of AoM, including Strategic Management, Organization and Management Theory, and Technology and Innovation Management. We also searched for unpublished studies in EBSCO, SSRN, and Google Scholar databases.	10 new studies, added	- This step addresses the “file drawer problem” (Rosenthal, 1995).
4: Identify if studies included the required statistical information for meta-analysis	Removed studies that did not report all the required information (e.g., sample size and correlations).	72 studies, removed	
5: Avoid double counting	Avoided double counting studies that referred to the same sample. For duplicated studies, we included only the most recently published ones in the final sample.	Three studies, removed	

Sample size: 205 empirical studies.

### 4.2. Coding

We hypothesized that the consideration of decision-makers' experience, domain expertise, compensation, and incentives influence the overall explanatory power of the performance feedback model. To test our hypotheses, we analyzed various studies in our sample based on performance feedback and decision-maker-level variables.

Regarding performance feedback mechanisms, we coded studies based on whether they analyzed the impact of performance below or above aspirations. For studies that analyzed responses to performance above aspirations, we also considered whether they show an increase in responses or a decrease in responses to performance above aspirations. This was determined by the correlation coefficient reported in each study: A positive coefficient for performance above aspirations indicates an increase in responses to performance above aspirations, and a negative coefficient for performance above aspirations indicates a decrease in responses to performance above aspirations.

Based on our hypotheses, we coded each research study based on the decision-maker-level variables that were analyzed, including job experience, domain expertise, incentives, and compensation. Table 2 provides an overview of our coding approach, including coding labels, examples of variables, and selected studies.

**Table 2:** Explanation of coding.

**Coding label**	**Examples of variables used**	**Sample papers**
Experience	CEO tenure, CEO experience	Arora and Dharwadkar, 2011; Kavadis and Castañer, 2015; Gomez-Mejia et al., 2018; Say and Vasudeva, 2020; Schumacher et al., 2020
Expertise	CEO specialization, CEO education	Baum et al., 2005; Wennberg and Holmquist, 2008; Mount and Baer, 2021; Wang and Zhang, 2021; Gaba et al., 2022
Incentives	CEO ownership, CEO stock options, CEO bonus	Harris and Bromiley, 2007; Shimizu, 2007; Alessandri, 2008; Arrfelt et al., 2012; Lim, 2018; He et al., 2021; Shi et al., 2022
Compensation	CEO salary, CEO compensation	Alessandri and Pattit, 2014; Lim, 2015, 2017; Ahn et al., 2020; Kolev and McNamara, 2020

To code job experience, we followed Gaba et al. (2022), considering a study as analyzing experience if it incorporated CEO career experience or tenure in its analyses. Similarly, for domain expertise, we followed Gaba et al. (2022) and coded a study as analyzing domain expertise if it examined CEO specialization or education. For incentives, we followed Harris and Bromiley (2007) and Lim (2017), coding a study as analyzing incentives if it incorporated CEO ownership, stock options, or bonus in its analyses. To indicate the presence or absence of these variables in each study, we used binary variables. In terms of compensation, we followed Lim and McCann (2014) and coded a study as analyzing compensation if it included CEO salary or pay in its analyses.

### 4.3. Analyses

To assess the overall effect sizes of performance below and above aspirations, we employed the bivariate meta-analytic procedure (Hunter and Schmidt, 1990) since this procedure is the most accurate and widely used method in management studies (Crook et al., 2008; Bergh et al., 2016). Using this procedure, we first calculated the sample size weighted average effect sizes from the Pearson correlation coefficients. This calculation was done using the following formula:


(1) $$r=\frac{\sum_{i}n_{i}r_{i}}{\sum_{i}n_{i}}$$

where *r* is the average effect size, n_i_ is the sample size, and r_i_ is the Pearson correlation coefficient for each study *i*. We used correlation coefficients to estimate effect sizes since they allow easy interpretation and limit downward bias (Geyskens et al., 2008; Aguinis et al., 2011). In general, we used all reported correlations from all studies in our sample to assess the overall effect sizes.

To test our hypotheses, we employed subgroup analyses, which is a suitable meta-analytic approach for categorical variables (Geyskens et al., 2008; Aguinis et al., 2011) ^3^. In these analyses, we created subgroups of studies based on two factors: the type of performance feedback mechanism (responses to performance below aspirations, increases in responses to performance above aspirations, and decreases in responses to performance above aspirations) and whether the studies included or excluded decision-makers' variables (experience, domain expertise, compensation, and incentives) in their analyses. We compared the effect sizes of these subgroups to determine whether the specific variable being studied had an impact on the analyzed relationship. We calculated the mean effect sizes for each subgroup and conducted Z-tests to assess differences across the groups (Schmidt and Hunter, 2014).

## 5. Results

### 5.1. Main results

Table 3 provides an overview of the overall findings regarding the relationships between performance feedback and organizational responses. Our analysis reveals that as performance decreases further below aspirations, organizational responses increase (*r* = −0.076, *p* = 0.000). Furthermore, our results indicate that the relationship between performance above aspirations and organizational response is not significant (*p* = 0.237). These results are in line with the prior meta-analyses on organizational performance feedback (Verver et al., 2019; Kotiloglu et al., 2021; Blettner et al., 2023).

**Table 3:** Meta-analysis results, baseline effects.

**Model**	**k**	**r**	**p**	**SE**	**CI 95%**	**Cr. I. 95%**
All responses to performance below aspirations	261	−0.076	0.000	0.006	−0.089; −0.067	−0.264; 0.111
All responses to performance above aspirations	225	0.011	0.237	0.009	−0.007; 0.029	−0.256; 0.278
Increase in responses to performance above aspirations	121	0.087	0.000	0.012	0.063; 0.111	−0.175; 0.349
Decrease in responses to performance above aspirations	104	−0.077	0.000	0.007	−0.091; −0.063	−0.206; 0.052

Number of data points (k), sample size weighted mean effect size (r), the standard deviation of sample size weighted correlation (SE), 95% confidence interval around the mean sample size weighted correlation (CI 95%), 95% credibility interval around the mean sample size weighted correlation (Cr. I. 95%), and Z-statistic (Z) for the critical ratio that indicates whether the subgroups are significantly different (significance of Z-test is determined using two-tailed tests).

Our theorizing differentiates between increases and decreases in responses to performance above aspirations. Accordingly, we reported the results for increases and decreases in responses to performance above aspirations separately. Effect sizes of increases (*r* = 0.087, *p* = 0.000) and decreases (*r* = −0.077, *p* = 0.000) in responses to performance above aspirations are statistically significant and practically meaningful.

Table 4 presents the results of the subgroup analyses on the effects of the decision-maker job experience. The results suggest that decision-maker job experience is associated with a weaker increase in responses to performance below aspirations; the effect size of performance below aspirations for studies that included the job experience variables (*r* = −0.059, *p*= 0.003) is smaller than the effect size of performance below aspirations for studies that excluded the job experience variables (*r* = −0.110, *p*= 0.000), and the difference is statistically significant (Δr = 0.051, *Z* = 2.319, *p* = 0.020). This result supports Hypothesis 1, which suggested that studies that include decision-makers' job experience show a weaker effect for responses to performance below aspirations than those that do not include experience.

**Table 4:** Meta-analysis results with experience variables (CEO tenure, experience).

**Criteria**	**Model**	**k**	**r**	**p**	**SE**	**CI 95%**	**Cr. I. 95%**	**Z**	**p_z_**
All responses to performance below aspirations	Models with experience variables	41	−0.059	0.003	0.020	−0.098; −0.020	−0.307; 0.189		
	Models without experience variables	51	−0.110	0.000	0.009	−0.127; −0.092	−0.218; −0.001	2.319	0.020
Increase in responses to performance above aspirations	Models with experience variables	17	0.088	0.063	0.047	−0.005; 0.181	−0.303; 0.479		
	Models without experience variables	24	0.117	0.003	0.039	0.039; 0.194	−0.260; 0.494	−0.466	0.641
Decrease in responses to performance above aspirations	Models with experience variables	18	−0.091	0.000	0.015	−0.120; −0.062	−0.203; 0.021		
	Models without experience variables	20	−0.091	0.000	0.012	−0.115; −0.067	−0.190; 0.009	−0.020	0.984

Number of data points (k), sample size weighted mean effect size (r), the standard deviation of sample size weighted correlation (SE), 95% confidence interval around the mean sample size weighted correlation (CI 95%), 95% credibility interval around the mean sample size weighted correlation (Cr. I. 95%), and Z-statistic (Z) for the critical ratio that indicates whether the subgroups are significantly different (significance of Z-test is determined using two-tailed tests).

Although we did not develop hypotheses on the influence of decision-maker job experience on the responses to performance above aspirations, our results indicate that decision-maker experience does not influence increases and decreases in responses to performance above aspirations. Specifically, the differences in the overall effects of studies that included or excluded experience variables are not statistically significant for increasing (*p* = 0.641) or decreasing (*p* = 0.984) responses to performance above aspirations.

Table 5 presents the results of the subgroup analyses on the effects of the decision-maker domain expertise. The results suggest that domain expertise strengthens the increase in responses to performance below aspirations; the overall effect of studies that included the domain expertise variables (*r* = −0.166, *p* = 0.000) is greater than the effect size of studies that excluded these variables (*r* = −0.078, *p* = 0.000). This difference is statistically significant (Δr = 0.088, *Z* = −4.272, *p*= 0.000), supporting Hypothesis 2, which posited that studies that include decision-makers' domain expertise show a stronger increase in responses to performance below aspirations than those that do not include domain expertise.

**Table 5:** Meta-analysis results with domain expertise variables (CEO specialization, education).

**Criteria**	**Model**	**k**	**r**	**p**	**SE**	**CI 95%**	**Cr. I. 95%**	**Z**	**p_z_**
All responses to performance below aspirations	Models with domain expertise variables	9	−0.166	0.000	0.017	−0.200; −0.132	−0.266; −0.067		
	Models without domain expertise variables	83	−0.078	0.000	0.011	−0.100; −0.056	−0.267; 0.111	−4.272	0.000
Increase in responses to performance above aspirations	Models with domain expertise variables	4	0.051	0.000	0.014	0.024; 0.078	0.007; 0.095		
	Models without domain expertise variables	35	0.116	0.001	0.035	0.048; 0.185	−0.287; 0.518	−1.726	0.084
Decrease in responses to performance above aspirations	Models with domain expertise variables	2	–	–	–	–	–		
	Models without domain expertise variables	38	−0.082	0.000	0.009	−0.100; −0.065	−0.180; 0.015	–	–

Number of data points (k), sample size weighted mean effect size (r), the standard deviation of sample size weighted correlation (SE), 95% confidence interval around the mean sample size weighted correlation (CI 95%), 95% credibility interval around the mean sample size weighted correlation (Cr. I. 95%), and Z-statistic (Z) for the critical ratio that indicates whether the subgroups are significantly different (significance of Z-test is determined using two-tailed tests).

Although we did not develop hypotheses on the influence of decision-makers' domain expertise on the responses to performance above aspirations, our results indicate that decision-makers' domain expertise does not have an impact on the increases and decreases in responses to performance above aspirations. The difference in the overall effects of studies that included or excluded experience variables is not statistically significant for increasing responses to performance above aspirations (*p* = 0.082). Moreover, we did not find enough empirical studies that reported decreases in responses to performance above aspirations and included the domain expertise variables (n = 2). As a result, we are unable to test the influence of decision-makers' domain expertise for decreases in responses to performance above aspirations.

Table 6 presents the results of the subgroup analyses on the effects of the decision-makers' performance-based incentives. These results indicate that decision-maker incentives do not have an impact on the increase in responses to performance below aspirations. The difference in the overall effects of studies that included or excluded inventive variables is not statistically significant for performance below aspirations (*p* = 0.395). Therefore, Hypothesis 3a, which proposed that studies that include decision-makers' performance-based incentives show a stronger increase in responses to performance below aspirations than those that do not include performance-based incentives, is not supported.

**Table 6:** Meta-analysis results with performance-based incentive variables (CEO ownership, options, bonus).

**Criteria**	**Model**	**k**	**r**	**p**	**SE**	**CI 95%**	**Cr. I. 95%**	**Z**	**p_z_**
All responses to performance below aspirations	Models with performance-based incentive variables	16	−0.072	0.000	0.019	−0.108; −0.035	−0.214; 0.071		
	Models without performance-based incentive variables	76	−0.091	0.000	0.012	−0.114; −0.067	−0.289; 0.107	0.850	0.395
Increase in responses to performance above aspirations	Models with performance-based incentive variables	8	0.028	0.020	0.012	0.004; 0.052	−0.027; 0.083		
	Models without performance-based incentive variables	33	0.122	0.001	0.037	0.050; 0.194	−0.290; 0.534	−2.431	0.015
Decrease in responses to performance above aspirations	Models with performance-based incentive variables	7	−0.049	0.000	0.010	−0.068; −0.029	−0.095; −0.003		
	Models without performance-based incentive variables	31	−0.102	0.000	0.011	−0.123; −0.081	−0.205; 0.001	3.648	0.000

Number of data points (k), sample size weighted mean effect size (r), the standard deviation of sample size weighted correlation (SE), 95% confidence interval around the mean sample size weighted correlation (CI 95%), 95% credibility interval around the mean sample size weighted correlation (Cr. I. 95%), and Z-statistic (Z) for the critical ratio that indicates whether the subgroups are significantly different (significance of Z-test is determined using two-tailed tests).

Our findings also reveal that decision-makers' performance-based compensation weakens both increasing and decreasing responses to performance above aspirations. For increasing responses to performance above aspirations, the effect is smaller in studies that included the incentive variables (*r* = 0.028, *p*= 0.020), compared to studies that excluded them (*r* = 0.122, *p* = 0.001). The difference between these two effects is statistically significant (Δr = 0.098, *Z* = −2.431, *p* = 0.015). Similarly, for decreasing responses to performance above aspirations, the effect is smaller in studies that included the incentive variables (*r* = −0.049, *p* = 0.000), compared to studies that excluded them (*r* = −0.102, *p* = 0.000). The difference between these two effects is statistically significant (Δr = 0.053, *Z* = 3.648, *p* = 0.000). These findings provide support for Hypothesis 3b, which posited that studies that include decision–makers' performance–based incentives show a weaker increase in responses to performance above aspirations than those that do not include performance-based incentives, and Hypothesis 3c, which suggested that studies that include decision-makers' performance-based incentives show a weaker decrease in responses to performance above aspirations than those that do not include performance-based incentives.

Table 7 presents the results of the subgroup analyses on the effects of the decision-makers' compensation. The findings suggest that decision-makers' compensation does not influence the increase in responses to performance below aspirations. Specifically, the difference in the overall effects of studies that included or excluded compensation variables is not statistically significant for performance below aspirations (*p* = 0.786). Therefore, Hypothesis 4a, which proposed that studies that include decision-makers' compensation show a stronger increase in responses to performance below aspirations than those that do not include compensation, is not supported.

**Table 7:** Meta-analysis results with compensation variables (CEO salary, compensation).

**Criteria**	**Model**	**k**	**r**	**p**	**SE**	**CI 95%**	**Cr. I. 95%**	**Z**	**p_z_**
All responses to performance below aspirations	Models with compensation variables	15	−0.093	0.000	0.021	−0.134; −0.051	−0.251; 0.066		
	Models without compensation variables	77	−0.086	0.000	0.012	−0.110; −0.063	−0.282; 0.110	−0.272	0.786
Increase in responses to performance above aspirations	Models with compensation variables	7	0.043	0.000	0.010	0.025; 0.061	0.008; 0.078		
	Models without compensation variables	34	0.118	0.001	0.036	0.048; 0.188	−0.290; 0.526	−2.050	0.040
Decrease in responses to performance above aspirations	Models with compensation variables	8	−0.085	0.000	0.016	−0.116; −0.055	−0.160; −0.010		
	Models without compensation variables	30	−0.093	0.000	0.011	−0.115; −0.071	−0.204; 0.018	0.400	0.689

Number of data points (k), sample size weighted mean effect size (r), the standard deviation of sample size weighted correlation (SE), 95% confidence interval around the mean sample size weighted correlation (CI 95%), 95% credibility interval around the mean sample size weighted correlation (Cr. I. 95%), and Z-statistic (Z) for the critical ratio that indicates whether the subgroups are significantly different (significance of Z-test is determined using two-tailed tests).

Furthermore, we found that decision-makers' compensation attenuates increases in responses to performance above aspirations. The effect of performance above aspirations on increases in responses is smaller in studies that included compensation variables (*r* = 0.043, *p* = 0.000), compared to studies that excluded them (*r* = 0.118, *p* = 0.001). The difference between these two effects is statistically significant (Δr = 0.075, *Z* = −2.050, *p* = 0.040). These findings support Hypothesis 4b, which suggested that studies that include decision-makers' compensation show a weaker increase in responses to performance above aspirations than those that do not include compensation. However, for decreasing responses to performance above aspirations, the difference between the effects of studies that included and excluded decision-makers' compensation variables is not statistically significant (*p* = 0.689). Therefore, Hypothesis 4c, which suggested that studies that include decision-makers' compensation show a weaker decrease in responses to performance above aspirations than those that do not include compensation, is not supported. Table 8 presents a summary of our hypotheses and findings.

**Table 8:** Summary of hypotheses and results.

**Hypothesis**	**Support**	**Results table**
H1: Studies that include decision-makers' job experience show a weaker increase in responses to performance below aspirations than those that do not include job experience.	Supported	Table 4
H2: Studies that include decision-makers' domain expertise show a stronger increase responses to performance below aspirations than those that do not include domain expertise.	Supported	Table 5
H3a: Studies that include decision-makers' performance-based incentives show a stronger increase in responses to performance below aspirations than those that do not include performance-based incentives.	Not supported	Table 6
H3b: Studies that include decision-makers' performance-based incentives show a weaker increase in responses to performance above aspirations than those that do not include performance-based incentives.	Supported	
H3c: Studies that include decision-makers' performance-based incentives show a weaker decrease in responses to performance above aspirations than those that do not include performance-based incentives.	Supported	
H4a: Studies that include decision-makers' compensation show a stronger increase in responses to performance below aspirations than those that do not include compensation.	Not supported	Table 7
H4b: Studies that include decision-makers' compensation show a weaker increase in responses to performance above aspirations than those that do not include compensation.	Supported	
H4c: Studies that include decision-makers' compensation show a weaker decrease in responses to performance above aspirations than those that do not include compensation.	Not supported	

### 5.2. Additional analyses

In addition to the variables hypothesized in our study, recent research by Blagoeva et al. (2020) and Gaba et al. (2022) has highlighted the significant role of decision-makers' overconfidence in shaping organizational responses to performance feedback. Specifically, Gaba et al. (2022) emphasize that decision-makers' experience plays a crucial role in their level of overconfidence. As a result, we also investigated the potential influence of decision-makers' overconfidence on the relationships between performance feedback and organizational responses. To conduct this analysis, we followed Blagoeva et al. (2020) and Schumacher et al. (2020), categorizing studies as examining overconfidence if they incorporated CEO tenure, gender, or bonus variables in their models.

Table 9 presents the results of the subgroup analyses on the effects of performance below and above aspirations on organizational responses, categorized by decision-maker overconfidence. Our findings indicate that decision-maker overconfidence weakens the increases in responses to performance below aspirations. In studies that included overconfidence variables, the effect of performance below aspirations (r = −0.067, *p* = 0.000) is smaller compared to studies that excluded these variables (r = −0.107, *p* = 0.001). The difference between these effects is statistically significant (Δr = 0.040, Z = 1.968, *p* = 0.049).

**Table 9:** Additional analysis, meta-analysis results with overconfidence variables (CEO tenure, gender, bonus).

**Criteria**	**Model**	**k**	**r**	**p**	**SE**	**CI 95%**	**Cr. I. 95%**	**Z**	**p_z_**
All responses to performance below aspirations	Models with overconfidence variables	46	−0.067	0.000	0.018	−0.103; −0.031	−0.306; 0.172		
	Models without overconfidence variables	46	−0.107	0.000	0.009	−0.126; −0.089	−0.220; 0.006	1.968	0.049
Increase in responses to performance above aspirations	Models with overconfidence variables	19	0.087	0.039	0.042	0.005; 0.170	−0.279; 0.454		
	Models without overconfidence variables	22	0.120	0.005	0.043	0.036; 0.204	−0.275; 0.515	−0.544	0.587
Decrease in responses to performance above aspirations	Models with overconfidence variables	20	−0.086	0.000	0.014	−0.113; −0.060	−0.194; 0.021		
	Models without overconfidence variables	18	−0.096	0.000	0.013	−0.121; −0.071	−0.195; 0.003	0.518	0.604

Number of data points (k), sample size weighted mean effect size (r), the standard deviation of sample size weighted correlation (SE), 95% confidence interval around the mean sample size weighted correlation (CI 95%), 95% credibility interval around the mean sample size weighted correlation (Cr. I. 95%), and Z-statistic (Z) for the critical ratio that indicates whether the subgroups are significantly different (significance of Z-test is determined using two-tailed tests).

Our results suggest that overconfidence is not associated with increases or decreases in responses to performance above aspirations as the differences between studies included and excluded overconfidence variables are not statistically significant (increases in responses: *p* = 0.587, decreases in responses: *p* = 0.604).

### 5.3. *Post hoc* analyses

To analyze whether outliers might have biased our results (Aguinis et al., 2010a; Schmidt and Hunter, 2014), we calculated the effect sizes for performance above and below aspirations without the outliers. Following Junni et al. (2013), we excluded correlation coefficients that were more than six standard deviations above or below the mean correlations of the overall sample. The results from this analysis are similar to the original results. Specifically, we found that, when potential outliers are excluded, the effect size of organizational performance feedback decreases by 0.001 for performance below aspirations and 0.007 for performance above aspirations. The difference between the two effect sizes is not significant (below: *p* = 0.949, above: *p* = 0.093). Moreover, when increases and decreases in responses to performance above aspirations are considered separately, when potential outliers are excluded, the effect size of organizational performance feedback decreases by 0.022 for increases in responses and 0.008 for decreases in responses. The difference between the two effect sizes is not significant (increases in responses: *p* = 0.144, decreases in responses: *p* = 0.446). The outlier analyses for subgroups were also insignificant.

To assess how many unpublished studies with null results would be needed to invalidate our results, we carried out the Fail-Safe N test (Rosenthal, 1995). The Fail-Safe N for the mean correlation between performance feedback and organizational actions is 1,097,455 for performance below aspirations and 22,679 for performance above aspirations. Moreover, when increases and decreases in responses to performance above aspirations are considered, the Fail-Safe N is 241,436 for increases in responses and 115,595 for decreases in responses to performance above aspirations. All Fail-Safe N values, including the ones for the subgroup analyses, exceeded the criterion suggested by Rosenthal (1979), i.e., five times the number of studies in the sample plus 10.

With a trim-and-fill analysis, we followed Aguinis et al. (2010b) to further assess the file drawer problem. The trim-and-fill method simulates studies that might be missing, and we included these simulated studies in our estimations of effect sizes (Duval and Tweedie, 2000). The estimated number of missing studies from our sample is zero, both for performance below and above aspirations. The estimated number of missing studies was also minimal for the subgroups (subgroups of studies that included and excluded individual-level variables), and the differences in our calculated effect sizes and the results of trim-and-fill methods were statistically insignificant.

To better assess whether publication bias exists, we followed Kromrey and Rendina-Gobioff (2006) and used Egger's regression method (Egger et al., 1997) and Begg's rank correlation method (Begg and Mazumdar, 1994), in addition to the trim-and-fill method to assess whether publication bias may have influenced our results. The results from these analyses were consistent for our main relationships and in line with the trim-and-fill analyses (performance below aspirations: Egger's test suggests that asymmetry in the funnel plot is not significant with p=0.573, and Begg's rank suggests that the funnel plot is not significantly asymmetric with p=0.866), increases in responses to performance above aspirations (Egger's test suggests that asymmetry in the funnel plot is not significant with *p* = 0.215, and Begg's rank suggests that the funnel plot is not significantly asymmetric with *p* = 0.105), decreases in responses to performance above aspirations (trim-and-fill analysis estimates 0 studies to be missing from the sample, Egger's test suggests that asymmetry in the funnel plot is not significant with p=0.295, and Begg's rank suggests that the funnel plot is not significantly asymmetric with p=0.368). Therefore, our analyses do not provide any evidence that publication bias exists in our sample.

As an additional analysis, we tested whether our subsamples that included and excluded the variables in question were significantly different in terms of their study characteristics, which could impact the extent of support for our hypotheses. We used meta-analytic regression models to examine the extent to which several methodological biases—i.e., publication year, publication quality, and research designs of studies—influenced the effect sizes of subgroup analyses. We ran these analyses between subgroups that included and excluded the experience, domain expertise, performance-based incentive, and compensation variables, and for increases in responses to performance below aspirations and increases and decreases in responses to performance above aspirations. We did not find significant differences between subsamples that included or excluded the variables in question in terms of methodological variables, except for publication year. This difference is likely because the studies that included the variables in question are more recently published, compared to the ones that excluded these variables. Overall, we did not observe any methodological biases between our subgroups.

## 6. Discussion

In this study, we meta-analytically examined the role of experience and incentives and how they relate to decision-making when decision-makers respond to organizational performance feedback. We find that both decision-makers' job experience and domain expertise influence their processing of feedback information below aspirations and incentives influence responses to performance feedback above aspirations.

### 6.1. Contributions

#### 6.1.1. Job experience versus domain expertise in performance feedback

Our analysis shows that decision-makers' job experience and domain expertise differ in their effects on responses to performance feedback. We argue that decision-makers' job experience derives from experiential learning that is prone to many biases, such as sampling bias, status quo bias, or attribution bias. Decision-makers overestimate the quality of the knowledge gained in this process: They believe that they are more competent in their role as the decision-maker than they objectively are. They become overconfident and, as a result, less responsive to performance feedback (Schumacher et al., 2020). Gaba et al. (2022) proposed overconfidence as a mechanism for the effect of experience on organizational responses. Our additional analyses demonstrate that overconfidence decreases responses across many diverse responses. We propose that the sources of overconfidence are rooted in the biases that arise from experiential learning.

We argue that decision-makers who have high domain expertise, however, are less prone to these biases because the knowledge is not acquired through experiential learning but by adopting codified knowledge of a field. This knowledge is more explicit and the rules that the learner derives are dissociated from the learner. This process of knowledge acquisition reduces biases and fosters broader information processing. Decision-makers with higher domain knowledge will become confident thanks to their knowledge base and engage in problemistic search in response to performance below aspirations. While Eggers and Suh (2019) theorized on domain-specific experience at the organizational level, our finding on decision-makers' domain expertise is novel to the Carnegie perspective. Our findings resonate with psychological studies that show a relationship between domain expertise and reduced loss aversion (see Mrkva et al., 2020 for a discussion).

#### 6.1.2. Explaining responses to performance below aspirations

Our diverging findings for job experience and domain expertise deepen our understanding of when decision-makers engage in problemistic search (Posen et al., 2018) and when they interpret performance feedback in a self-enhancing way (Audia and Brion, 2007; Jordan and Audia, 2012; Lim and Audia, 2020). We theorized that experiential learning is associated with many biases such as attribution bias (Alicke and Sedikides, 2009) that lead to self-enhancing interpretation of performance feedback and instill overconfidence in decision-makers. We conclude that problemistic search may be reduced by individual decision-makers' information processing which is more biased for job experience than for domain expertise.

#### 6.1.3. Incentives and compensation in performance feedback

We contribute to the theorizing of the effect of incentives and compensation in the performance feedback mechanism. We find that incentives/compensation influence responses above aspirations. They render the aspiration level more salient, increasing decision-makers' focus on the aspiration level. Incentives, in particular, make decision-makers less complacent but also less ambitious. This is in line with Lim and McCann (2014) who showed decreased risk-taking for performance above aspirations. Thanks to our differentiation between increases and decreases of responses to performance above aspirations that is not commonly made in the literature, we were able to detect more nuanced effects; specifically, we are able to attribute this effect to reduced ambition rather than complacency. While both incentives and compensation emphasize the aspiration level, incentives are related to continued, moderate risk-taking above aspirations while compensation is not.

BAM predicts incentives and compensation increase responses below aspirations, but we did not find such evidence. Our non-findings for incentives and compensation for performance aspirations are also contrary to Lim and McCann (2014) who showed that CEOs who receive high incentives (here: stock options) become risk averse. This could be because decision-makers are already intrinsically motivated to search for solutions to their organizations' performance problems (Greve, 2003). Another reason is that decision-makers who receive high incentives are powerful and able to embellish the outcomes of their decisions, for instance, by switching reference groups (Audia et al., 2022). Our non-findings also resonate with Hogarth et al. (1991) who find incentives to be ineffective in situations of negative feedback. Similarly, Etchart-Vincent and l'Haridon (2010) found that incentives were crucial in the gain domain but not in the loss domain. Therefore, our meta-analytic results are consistent with several relevant psychological studies. We show that these results—that are generally generated by individuals in the laboratory—apply to the context of organizations.

#### 6.1.4. Explaining responses to performance above aspirations

We contribute to the discussion concerning controversy on responses to performance above aspirations (Kotiloglu et al., 2021). Scholars have proposed organizational factors such as organizational size and slack (e.g., Singh, 1986; Greve, 2003) as a potential explanation for why increases in responses to performance above aspirations are observed in some organizations and contexts, but decreases in responses are observed in others, but the underlying mechanism and conditions for when firms increase their responses to performance above aspirations are not yet well understood (Ref and Shapira, 2017). This study makes clear that incentives and compensation, inasmuch as they affect the decision-makers' motivation to take risk, influence their individual responses. We believe that the specific incentive mixes which decision-makers receive will determine whether they activate organizational slack in the first place. Ignoring the motivation of decision-makers or assuming that all decision-makers are equally motivated, independent of their specific situation (e.g., incentives), is not an adequate reflection of what we now know.

### 6.2. Practical implications of our results

Our results have important implications for practice. Since executives' experience affects responses to feedback, it is important to carefully screen executives' profiles during the selection and hiring processes. They are also relevant in executive development, in terms of raising awareness of the differences and levels of rigidity in individuals' cognitive frames through specialized training.

Our findings on incentives imply that organizational policy needs to create appropriate and adaptive incentives and compensation packages for executives. It is important to balance the advantages and drawbacks of increasing incentive: An increase in executives' incentives may decrease their intrinsic motivation (Wiersema, 1992; Deci et al., 1999); decision-makers may become overly focused on their high pay reference points (Pokorny, 2008) and, as a result, become less interested in learning from feedback (Hogarth et al., 1991). Incentives lead to less risk-taking when performance is above aspirations.

### 6.3. Limitations and future research

Our study is limited by the samples used in the underlying studies. Most empirical BTOF studies are based on larger, publicly traded companies. There are many constraints and specific regulations, for example, shareholder expectations and performance reporting standards for individuals, in large, public organizations. Therefore, some variables representing the individual level might not show sufficient heterogeneity. This could lead to lower effect sizes. We expect a stronger effect size for individual-level variables in samples consisting of smaller or private companies for which systematic data are not generally available. Given that the CEO effect (on performance) in general has increased over the past years (Quigley and Hambrick, 2015; Quigley and Graffin, 2017), we also expect that this effect will get stronger in BTOF studies as well.

There are several important differences among cultures, such as risk preferences and uncertainty avoidance, which are relevant for responses to performance feedback (Hofstede, 2001; Statman, 2008; Kotiloglu et al., 2023). However, most empirical BTOF studies are based on samples from the United States. This leads to more homogeneity than is representative.

While our analysis cumulatively accounted for context factors such as riskiness, factors related to the industry and economic environment and our findings are generalizable across context. While this allows us to make generalizable predictions, it does not allow us to dissect facets of contextual factors. For instance, the ambiguity and the riskiness of context are likely to affect this relationship (Audia and Brion, 2007; Gächter et al., 2022). As more BTOF studies become available, a meta-analysis of the effect of those relevant context factors will be possible. Similarly, it will be interesting to differentiate different types of organizational responses (Kuusela et al., 2017) and diverse and diverging performance measures (Audia and Brion, 2007; Steinberg et al., 2022).

We also believe that job experience/domain expertise and incentives/compensation will affect which reference points decision-makers consider meaningful and against which they assess their own performance (Audia et al., 2015, 2022). Tarakci et al. (2018) showed that decision-makers use their individual performance feedback as a reference point in addition to organizational feedback. Individuals may have reference points within and outside of the organizations (Kacperczyk et al., 2015). March (1994, p. 31) already hinted at such individual-based reference points, writing that aspirations “... are affected by the past performances of the particular individual or organizations and by the past performance of those individuals and organizations perceived as comparable”. Building on March's statement, future models of organizational feedback could consider two individual-level reference points (individual feedback relative to own prior performance and individual feedback relative to peers) in addition to the two organizational-level reference points (organizational performance relative to own prior performance and organizational performance relative to peer organizations) that are typically considered. Similarly, decision-makers with different cognitive frames will likely have idiosyncratic reference points that go beyond the standard performance feedback model (Audia et al., 2022). It will be important to study how decision-makers balance their attention among multiple reference points (Hu et al., 2017; Tarakci et al., 2018) and how their attention allocation to diverse reference points mediates the relationship between performance feedback and responses.

Since we identified overconfidence as a mechanism that influences whether firms increase their responses to performance below aspirations or not, we propose that future studies examine this construct more closely. Scholars may opt for experimental, survey-based, or text-based approaches. Building on Schumacher et al. (2020)'s work that illustrated the relevance of overconfidence using a media-based and an option-based measure of overconfidence, it will be important to further explore overconfidence in the performance feedback mechanism using direct measures of the construct. Scholars may measure overconfidence as miscalibration (Russo and Schoemaker, 1992), as decision behavior (Glaser and Weber, 2007), or perform psycholinguistic analyses of decision-makers' text or speech (Pennebaker et al., 2015; Zyung and Shi, 2022). Since these measures capture different facets of the construct, robustness tests of alternative measures are crucial. We also hope that researchers will examine overconfidence in diverse contexts as it can vary across task environments (Glaser et al., 2005).

## 7. Conclusion

Within the Carnegie perspective, BTOF explains organizational decision-making. While it proposes that decisions are made by individual managers, the theory has, as we allude to, unfinished business. BTOF scholars only recently started analyzing the role of individual decision-makers in organizational decision-making. There is more work to be done regarding the integration of the individual level to the organizational decision-making process. Our meta-analytic review showed that individual decision-makers' job experience and domain expertise influence organizational responses to performance below aspirations and performance-based incentives and compensation influence responses to performance above aspirations. In doing so, we open multiple pathways and opportunities for future studies that seek to extend the BTOF by further exploring specific individual-level factors. We believe that there is great promise for the insights and contributions of scholars in the field of psychology to enrich the theorizing of the role of individual decision-makers in the Carnegie perspective.

## Data availability statement

The raw data supporting the conclusions of this article will be made available by the authors, without undue reservation.

## Author contributions

All authors listed have made a substantial, direct, and intellectual contribution to the work and approved it for publication.

## Footnotes

^1^There are two editions of Cyert and March's book “A Behavioral Theory of the Firm”, namely the original edition from 1963 and a new edition from 1992. While the two editions are very similar, we are using the 1992 version in our study for consistency.

^2^Bibliographic details for all studies can be found in Appendix.

^3^In addition to subgroup analyses, meta-regression analysis is another approach that allows testing for the interaction effects of the variables in question. However, subgroup analyses are more appropriate to test for the interaction effect of binary and categorical variables, while meta-regression analyses are more appropriate for continuous variables (Aguinis et al., 2011). Therefore, we opted for subgroup analyses to test our hypotheses.

## References

[B1] Aguinis H. Dalton D. R. Bosco F. A. Pierce C. A. Dalton C. M. (2010a). Meta-analytic choices and judgment calls: Implications for theory building and testing, obtained effect sizes, and scholarly impact. J. Manage. 37, 5–38. 10.1177/0149206310377113

[B2] Aguinis H. Gottfredson R. K. Wright T. A. (2011). Best-practice recommendations for estimating interaction effects using meta-analysis. J. Organ. Behav. 32, 1033–1043. 10.1002/job.719

[B3] Aguinis H. Pierce C. A. Bosco F. A. Dalton D. R. Dalton C. M. (2010b). Debunking myths and urban legends about meta-analysis. Organ. Res. Methods 14, 306–331. 10.1177/1094428110375720

[B4] Aguinis H. Ramani R. S. Alabduljader N. (2018). What you see is what you get? Enhancing methodological transparency in management research. Acad. Manag. Ann. 12, 83–110. 10.5465/annals.2016.0011

[B5] Ahn S. Cho C. K. Cho T. S. (2020). Performance feedback and organizational learning: the role of regulatory focus. Manag. Decis. 59, 616–637. 10.1108/MD-09-2019-1319

[B6] Alessandri T. M. (2008). Risk and procedural rationality: a behavioral theory perspective. J. Strategy Manag. 1, 198–217. 10.1108/17554250810926375

[B7] Alessandri T. M. Pattit J. M. (2014). Drivers of R&D investment: the interaction of behavioral theory and managerial incentives. J. Bus. Res. 67, 151–158. 10.1016/j.jbusres.2012.11.001

[B8] Alicke M. D. Sedikides C. (2009). Self-enhancement and self-protection: what they are and what they do. Eur. Rev. Social Psychol. 20, 1–48. 10.1080/1046328080261386635875947

[B9] Argote L. (1999). Organizational Learning: Creating, Retaining, and Transferring Knowledge. Boston: Kluwer Academic.

[B10] Arora P. Dharwadkar R. (2011). Corporate governance and corporate social responsibility (CSR): The moderating roles of attainment discrepancy and organization slack. Corp. Govern. 19, 136–152. 10.1111/j.1467-8683.2010.00843.x

[B11] Arrfelt M. Wiseman R. M. Hult G. T. M. (2012). Looking backward instead of forward: Aspiration-driven influences on the efficiency of the capital allocation process. Acad. Manage. J. 56, 1081–1103. 10.5465/amj.2010.0879

[B12] Audia P. G. Brion S. (2007). Reluctant to change: self-enhancing responses to diverging performance measures. Organ. Behav. Hum. Decis. Process. 102, 255–269. 10.1016/j.obhdp.2006.01.007

[B13] Audia P. G. Brion S. Greve H. R. (2015). Self-assessment, self-enhancement, and the choice of comparison organizations for evaluating organizational performance. Cognit. Strat. 32, 89–118. 10.1108/S0742-332220150000032018

[B14] Audia P. G. Greve H. R. (2006). Less likely to fail: low performance, firm size, and factory expansion in the shipbuilding industry. Manage. Sci. 52, 83–94. 10.1287/mnsc.1050.0446

[B15] Audia P. G. Greve H. R. (2021). “Organizational Learning from Performance Feedback: A Behavioral Perspective on Multiple Goals,” in Elements in Organization Theory Series. (Cambridge, UK: Cambridge University Press). 10.1017/9781108344289

[B16] Audia P. G. Locke E. A. Smith K. G. (2000). The paradox of success: an archival and a laboratory study of strategic persistence following radical environmental change. Acad. Manage. J. 43, 837–853. 10.2307/1556413

[B17] Audia P. G. Rousseau H. E. Brion S. (2022). CEO power and nonconforming reference group selection. Organiz. Sci. 10.1287/orsc.2020.1397

[B18] Baum J. A. Rowley T. J. Shipilov A. V. Chuang Y.-T. (2005). Dancing with strangers: Aspiration performance and the search for underwriting syndicate partners. Adm. Sci. Q. 50, 536–575. 10.2189/asqu.50.4.536

[B19] Begg C. B. Mazumdar M. (1994). Operating characteristics of a rank correlation test for publication bias. Biometrics. 50, 1088–1101. 10.2307/2533446 7786990

[B20] Bergh D. D. Aguinis H. Heavey C. Ketchen D. J. Boyd B. K. Su P.. (2016). Using meta-analytic structural equation modeling to advance strategic management research: Guidelines and an empirical illustration via the strategic leadership-performance relationship. Strateg. Manag. J. 37, 477–497. 10.1002/smj.2338

[B21] Bilgili T. V. Calderon C. J. Allen D. G. Kedia B. L. (2016). Gone with the wind: A meta-analytic review of executive turnover, its antecedents, and postacquisition performance. J. Manage. 43, 1966–1997. 10.1177/0149206316635252

[B22] Blagoeva R. R. Mom T. J. Jansen J. J. George G. (2020). Problem-solving or self-enhancement? A power perspective on how CEOs affect R&D search in the face of inconsistent feedback. Acad. Manage. J. 63, 332–355. 10.5465/amj.2017.0999

[B23] Blettner D. Kotiloglu S. Lechler T. (2019). Variations in the effects of performance above aspirations: Empirical artifact or theoretical gap? Acad. Manage. J. 2019, 12248. 10.5465/AMBPP.2019.12248abstract

[B24] Blettner D. Kotiloglu S. Lechler T. G. (2023). Self-assessment versus self-improvement motives: how does social reference group selection influence organizational responses to performance feedback? Br. J. Management. 10.1111/1467-8551.12700

[B25] Chatterjee A. Hambrick D. C. (2011). Executive personality, capability cues, and risk taking. Adm. Sci. Q. 56, 202–237. 10.1177/0001839211427534

[B26] Chen W.-R. Miller K. D. (2007). Situational and institutional determinants of firms' R&D search intensity. Strateg. Manag. J. 28, 369–381. 10.1002/smj.594

[B27] Combs J. G. Crook T. R. Rauch A. (2018). Meta-analytic research in management: Contemporary approaches, unresolved controversies, and rising standards. J. Manag. Stud. 56, 1–18. 10.1111/joms.12427

[B28] Crook T. R. Ketchen D. J. Combs J. G. Todd S. Y. (2008). Strategic resources and performance: a meta-analysis. Strateg. Manag. J. 29, 1141–1154. 10.1002/smj.703

[B29] Cyert R. M. March J. G. (1963). A Behavioral Theory of the Firm. Englewood Cliffs, NJ: Prentice-Hall, Inc.

[B30] Cyert R. M. March J. G. (1992). A Behavioral Theory of the Firm. Malden, MA: Blackwell Publishers.

[B31] Deci E. L. Koestner R. Ryan R. M. (1999). A meta-analytic review of experiments examining the effects of extrinsic rewards on intrinsic motivation. Psychol. Bull. 125, 627–658. 10.1037/0033-2909.125.6.627 10589297

[B32] Devers C. E. Cannella A. A. Reilly G. P. Yoder M. E. (2016). Executive compensation: a multidisciplinary review of recent developments. J. Manage. 33, 1016–1072. 10.1177/0149206307308588

[B33] D'Oria L. Crook T. R. Ketchen D. J. Sirmon D. G. Wright M. (2021). The evolution of resource-based inquiry: a review and meta-analytic integration of the strategic resources–actions–performance pathway. J. Manage. 47, 1383–1429. 10.1177/0149206321994182

[B34] Duval S. J. Tweedie R. L. (2000). Trim and fill: a simple funnel plot-based method of testing and adjusting for publication bias in meta-analysis. Biometrics. 56, 455–463. 10.1111/j.0006-341X.2000.00455.x 10877304

[B35] Egger M. Smith G. D. Schneider M. Minder C. (1997). Bias in meta-analysis detected by a simple, graphical test. BMJ. 315, 629–634. 10.1136/bmj.315.7109.629 9310563 PMC2127453

[B36] Eggers J. P. Suh J. H. (2019). Experience and behavior: how negative feedback in new versus experienced domains affects firm action and subsequent performance. Acad. Manage. J. 62, 309–334. 10.5465/amj.2017.0046

[B37] Ert E. (2012). On the value of experience-based decisions in studying constructs of risk taking. Front. Psychol. 3, 7. 10.3389/fpsyg.2012.00007 22291679 PMC3265759

[B38] Etchart-Vincent N. l'Haridon O. (2010). Monetary incentives in the loss domain and behavior toward risk: an experimental comparison of three reward schemes including real losses. J. Risk Uncertain. 42, 61–83. 10.1007/s11166-010-9110-0

[B39] Finkelstein S. Hambrick D. C. (1990). Top-management-team tenure and organizational outcomes: The moderating role of managerial discretion. Adm. Sci. Q. 35, 484–503. 10.2307/2393314

[B40] Fox C. R. Hadar L. (2006). “Decisions from experience” = sampling error + prospect theory: Reconsidering Hertwig, Barron, Weber & Erev (2004). Judgm. Decis. Mak. 1, 159–161. 10.1017/S1930297500002370

[B41] Gaba V. Lee S. Meyer-Doyle P. Zhao-Ding A. (2022). Prior experience of managers and maladaptive responses to performance feedback: evidence from mutual funds. Organiz. Sci. 34, 509–986. 10.1287/orsc.2022.1605

[B42] Gächter S. Johnson E. J. Herrmann A. (2022). Individual-level loss aversion in riskless and risky choices. Theory Decis. 92, 599–624. 10.1007/s11238-021-09839-8

[B43] Gavetti G. Levinthal D. Ocasio W. (2007). Perspective—neo-carnegie: the carnegie school's past, present, and reconstructing for the future. Organiz. Sci. 18, 523–536. 10.1287/orsc.1070.0277

[B44] Geyskens I. Krishnan R. Steenkamp J.-B. E. M. Cunha P. V. (2008). A review and evaluation of meta-analysis practices in management research. J. Manage. 35, 393–419. 10.1177/0149206308328501

[B45] Glaser M. Langer T. Weber M. (2005). Overconfidence of Professionals and Lay Men : Individual Differences Within and Between Tasks?. Mannheim: Universitat Mannheim. 10.1037/e722842011-018

[B46] Glaser M. Weber M. (2007). Why inexperienced investors do not learn: They do not know their past portfolio performance. Finance Res. Lett. 4, 203–216. 10.1016/j.frl.2007.10.001

[B47] Glockner A. Pachur T. (2012). Cognitive models of risky choice: parameter stability and predictive accuracy of prospect theory. Cognition. 123, 21–32. 10.1016/j.cognition.2011.12.002 22226615

[B48] Gomez-Mejia L. R. Patel P. C. Zellweger T. M. (2018). In the horns of the dilemma: Socioemotional wealth, financial wealth, and acquisitions in family firms. J. Manage. 44, 1369–1397. 10.1177/0149206315614375

[B49] Gomez-Mejia L. R. Wiseman R. M. (1997). Reframing executive compensation. An assessment and outlook. J. Manage. 23, 291–374. 10.1016/S0149-2063(97)90035-0

[B50] Greve H. R. (1998). Performance, aspirations, and risky organizational change. Adm. Sci. Q. 43, 58–86. 10.2307/2393591

[B51] Greve H. R. (2003). Organizational Learning from Performance Feedback: A Behavioral Perspective on Innovation and Change. Cambridge, UK: Cambridge University Press. 10.1017/CBO9780511615139

[B52] Harris J. Bromiley P. (2007). Incentives to cheat: the influence of executive compensation and firm performance on financial misrepresentation. Organiz. Sci. 18, 350–367. 10.1287/orsc.1060.0241

[B53] He L. Huang L. Yang G. (2021). Invest in innovation or not? How managerial cognition and attention allocation shape corporate responses to performance shortfalls. Manage. Organizat. Rev. 17, 815–850. 10.1017/mor.2021.58

[B54] Hofstede G. (2001). Culture's Consequences, Comparing Values, Behaviors, Institutions, and Organizations Across Nations. Thousand Oaks, CA: Sage.

[B55] Hogarth R. M. Gibbs B. J. McKenzie C. R. Marquis M. A. (1991). Learning from feedback: exactingness and incentives. J. Experim. Psychol. 17, 734–752. 10.1037/0278-7393.17.4.734 1832435

[B56] Hönl A. Meissner P. Wulf T. (2017). Risk attribution theory: an exploratory conceptualization of individual choice under uncertainty. J. Behav. Experim. Econ. 67, 20–27. 10.1016/j.socec.2017.02.001

[B57] Hu S. He Z. -L. Blettner D. P. Bettis R. A. (2017). Conflict inside and outside: Social comparisons and attention shifts in multidivisional firms. Strateg. Manag. J. 38, 1435–1454. 10.1002/smj.2556

[B58] Hunter J. E. Schmidt F. L. (1990). Methods of Meta-Analysis. Newbury Park, CA.: Sage.

[B59] Jordan A. H. Audia P. G. (2012). Self-enhancement and learning from performance feedback. Acad. Manage. Rev. 37, 211–231. 10.5465/amr.2010.0108

[B60] Junni P. Sarala R. M. Taras V. Tarba S. Y. (2013). Organizational ambidexterity and performance: a meta-analysis. Acad. Manage. Perspectives 27, 299–312. 10.5465/amp.2012.0015

[B61] Kacperczyk A. Beckman C. M. Moliterno T. P. (2015). Disentangling risk and change: Internal and external social comparison in the mutual fund industry. Adm. Sci. Q. 60, 228–262. 10.1177/0001839214566297

[B62] Kahneman D. (2011). Thinking, Fast and Slow. Chicago, IL: Macmillan.

[B63] Kahneman D. Tversky A. (1979). Prospect theory: an analysis of decision under risk. Econometrica. 263–291. 10.2307/1914185

[B64] Kavadis N. Castañer X. (2015). Who drives corporate restructuring? Co-Existing owners in French firms. Corporate Govern. 23, 417–433. 10.1111/corg.12108

[B65] Kluger A. N. DeNisi A. (1996). The effects of feedback interventions on performance: a historical review, a meta-analysis, and a preliminary feedback intervention theory. Psychol. Bull. 119, 254–284. 10.1037/0033-2909.119.2.254

[B66] Kolev K. D. McNamara G. (2020). The role of top management teams in firm responses to performance shortfalls. Strategic Organiz. 20, 3. 10.1177/1476127020962683

[B67] Kotiloglu S. Blettner D. Lechler T. G. (2023). Integrating national culture into the organizational performance feedback theory. Eur. Manag. J. 10.1016/j.emj.2023.01.003 PMC10354647 37476085

[B68] Kotiloglu S. Chen Y. Lechler T. (2021). Organizational responses to performance feedback: A meta-analytic review. Strategic Organiz. 19, 285–311. 10.1177/1476127019883361

[B69] Kraatz M. S. Zajac E. J. (2001). How organizational resources affect strategic change and performance in turbulent environments: theory and evidence. Organiz. Sci. 12, 632–657. 10.1287/orsc.12.5.632.10088

[B70] Kromrey J. D. Rendina-Gobioff G. (2006). On knowing what we do not know: An empirical comparison of methods to detect publication bias in meta-analysis. Educ. Psychol. Meas. 66, 357–373. 10.1177/0013164405278585

[B71] Kuusela P. Keil T. Maula M. (2017). Driven by aspirations, but in what direction? Performance shortfalls, slack resources, and resource-consuming vs. resource-freeing organizational change. Strateg. Manag. J. 38, 1101–1120. 10.1002/smj.2544

[B72] Larraza-Kintana M. Wiseman R. M. Gomez-Mejia L. R. Welbourne T. M. (2007). Disentangling compensation and employment risks using the behavioral agency model. Strateg. Manag. J. 28, 1001–1019. 10.1002/smj.624

[B73] Levinthal D. A. March J. G. (1993). The myopia of learning. Strateg. Manag. J. 9, 95–112. 10.1002/smj.4250141009

[B74] Levitt B. March J. G. (1988). Organizational learning. Annu. Rev. Sociol. 14, 319–340. 10.1146/annurev.so.14.080188.001535

[B75] Lim E. (2015). The role of reference point in CEO restricted stock and its impact on R&D intensity in high-technology firms. Strateg. Manag. J. 36, 872–889. 10.1002/smj.2252

[B76] Lim E. (2017). CEO option wealth and firm risk-taking: an analysis of multiple reference points. Long Range Plann. 50, 809–825. 10.1016/j.lrp.2016.12.013

[B77] Lim E. (2018). Social pay reference point, external environment, and risk taking: an integrated behavioral and social psychological view. J. Bus. Res. 82, 68–78. 10.1016/j.jbusres.2017.08.001

[B78] Lim E. Audia P. G. (2020). Problem-solving or self-enhancing? Influences of diversification and bright spot on corporate resource allocation responses to performance shortfalls. Strategy Sci. 5, 348–368. 10.1287/stsc.2020.0117

[B79] Lim E. McCann B. T. (2014). Performance feedback and firm risk taking: the moderating effects of CEO and outside director stock options. Organiz. Sci. 25, 262–282. 10.1287/orsc.2013.0830

[B80] March J. G. (1991). Exploration and exploitation in organizational learning. Organiz. Sci. 2, 71–87. 10.1287/orsc.2.1.71

[B81] March J. G. (1994). Primer on Decision Making: How Decisions Happen. New York, NY: Simon and Schuster.

[B82] March J. G. (2008). Explorations in Organizations. Stanford, CA: Stanford University Press. 10.1515/9781503627147

[B83] March J. G. (2010). The Ambiguities of Experience. Ithaca, NY: Cornell University Press. 10.7591/9780801459016

[B84] March J. G. Shapira Z. (1992). Variable risk preferences and the focus of attention. Psychol. Rev. 99, 172–183. 10.1037/0033-295X.99.1.172

[B85] March J. G. Simon H. A. (1958). Organizations. New York: John Wiley & Sons.

[B86] Miller D. (1991). Stale in the saddle: CEO tenure and the match between organization and environment. Manage. Sci. 37, 34–52. 10.1287/mnsc.37.1.34

[B87] Mount M. P. Baer M. (2021). CEOs' regulatory focus and risk-taking when firms perform below and above the bar. J. Manage. 10.1177/01492063211016029

[B88] Mrkva K. Johnson E. J. Gächter S. Herrmann A. (2020). Moderating loss aversion: loss aversion has moderators, but reports of its death are greatly exaggerated. J. Cons. Psychol. 30, 407–428. 10.1002/jcpy.1156

[B89] Nohria N. Gulati R. (1996). Is slack good or bad for innovation? Acad. Manage. J. 39, 1245–1264. 10.5465/256998

[B90] Pachur T. Bröder A. Marewski J. N. (2008). The recognition heuristic in memory-based inference: is recognition a non-compensatory cue? J. Behav. Decis. Mak. 21, 183–210. 10.1002/bdm.581

[B91] Pachur T. Mata R. Hertwig R. (2017). Who dares, who errs? Disentangling cognitive and motivational roots of age differences in decisions under risk. Psychol. Sci. 28, 504–518. 10.1177/0956797616687729 28406375

[B92] Pennebaker J. W. Boyd R. L. Jordan K. Blackburn K. (2015). The Development and Psychometric Properties of LIWC2015. Austin, TX: University of Texas at Austin.

[B93] Pokorny K. (2008). Pay—but do not pay too much. J. Econ. Behav. Organ. 66, 251–264. 10.1016/j.jebo.2006.03.007

[B94] Posen H. E. Keil T. Kim S. Meissner F. (2018). Renewing research on problemistic search - a review and research agenda. Acad. Manag. Ann. 12, 208–251. 10.5465/annals.2016.0018

[B95] Quigley T. J. Graffin S. D. (2017). Reaffirming the CEO effect is significant and much larger than chance: a comment on Fitza (2014). Strateg. Manag. J. 38, 793–801. 10.1002/smj.2503

[B96] Quigley T. J. Hambrick D. C. (2015). Has the “CEO effect” increased in recent decades? A new explanation for the great rise in America's attention to corporate leaders. Strateg. Manag. J. 36, 821–830. 10.1002/smj.2258

[B97] Ref O. Shapira Z. (2017). Entering new markets: the effect of performance feedback near aspiration and well below and above it. Strateg. Manag. J. 38, 1416–1434. 10.1002/smj.2561

[B98] Rosenthal R. (1979). The “file drawer problem” and tolerance for null results. Psychol. Bull. 86, 638–641. 10.1037/0033-2909.86.3.638

[B99] Rosenthal R. (1995). Writing meta-analytic reviews. Psychol. Bull. 118, 183–192. 10.1037/0033-2909.118.2.183

[B100] Russo J. E. Schoemaker P. J. (1992). Managing overconfidence. Sloan Manage. Rev. 33, 7–17.10120627

[B101] Say G. Vasudeva G. (2020). Learning from digital failures? The effectiveness of firms' divestiture and management turnover responses to data breaches. Strat. Sci. 5, 117–142. 10.1287/stsc.2020.0106

[B102] Schmidt F. L. Hunter J. E. (2014). Methods of Meta-Analysis: Correcting Error and Bias in Research Findings. Newbury Park, CA.: Sage. 10.4135/9781483398105

[B103] Schumacher C. Keck S. Tang W. (2020). Biased interpretation of performance feedback: the role of CEO overconfidence. Strateg. Manag. J. 41, 1139–1165. 10.1002/smj.3138

[B104] Sedikides C. Strube M. J. (1997). “Self evaluation: To thine own self be good, to thine own self be sure, to thine own self be true, and to thine own self be better,” in Advances in Experimental Social Psychology, Zanna, M. P. Cambridge, MA: Academic Press. p. 209–269. 10.1016/S0065-2601(08)60018-0

[B105] Shi W. Chen G. Li B. (2022). Problem solving or responsibility avoidance? The role of CEO internal attribution tendency in shaping corporate downsizing in response to performance shortfalls. J. Manag. Stud. 10.1111/joms.12896

[B106] Shimizu K. (2007). Prospect theory, behavioral theory, and the threat-rigidity thesis: combinative effects on organizational decisions to divest formerly acquired units. Acad. Manage. J. 50, 1495–1514. 10.5465/amj.2007.28226158

[B107] Shinkle G. A. (2012). Organizational aspirations, reference points, and goals: Building on the past and aiming for the future. J. Manage. 38, 415–455. 10.1177/0149206311419856

[B108] Simon H. A. (1947). Administrative Behavior: A Study of Decision-Making Processes in Administrative Organization. New York: The Macmillan Company.

[B109] Singh J. V. (1986). Performance, slack, and risk taking in organizational decision making. Acad. Manage. J. 29, 562–585. 10.2307/256224

[B110] Statman M. (2008). Countries and culture in behavioral finance. CFA Inst. Conf. Proc. Quart. 5, 38–44. 10.2469/cp.v25.n3.6

[B111] Steinberg P. J. Asad S. Lijzenga G. (2022). Narcissistic CEOs' dilemma: the trade-off between exploration and exploitation and the moderating role of performance feedback. J. Prod. Innovat. Manage. 39, 773–796. 10.1111/jpim.12644

[B112] Tarakci M. Ate,ş N. Y. Floyd S. W. Ahn Y. Wooldridge B. (2018). Performance feedback and middle managers' divergent strategic behavior: the roles of social comparisons and organizational identification. Strateg. Manag. J. 39, 1139–1162. 10.1002/smj.2745

[B113] Trepel C. Fox C. R. Poldrack R. A. (2005). Prospect theory on the brain? Toward a cognitive neuroscience of decision under risk. Brain Res Cogn Brain Res 23, 34–50. 10.1016/j.cogbrainres.2005.01.016 15795132

[B114] Vanneste B. S. Puranam P. Kretschmer T. (2014). Trust over time in exchange relationships: meta-analysis and theory. Strateg. Manag. J. 35, 1891–1902. 10.1002/smj.2198

[B115] Verver H. van Zelst M. Lucas G. J. M. Meeus M. T. H. (2019). Understanding Heterogeneity in the Performance Feedback –Organizational Responsiveness Relationship: A Meta-Analysis. Available online at: https://osf.io/hq4uw. 10.31219/osf.io/hq4uw

[B116] Wang K. Zhang X. (2021). The effect of media coverage on disciplining firms' pollution behaviors: evidence from Chinese heavy polluting listed companies. J. Clean. Prod. 280, 123035. 10.1016/j.jclepro.2020.123035

[B117] Wangrow D. B. Kolev K. Hughes-Morgan M. (2019). Not all responses are the same: how CEO cognitions impact strategy when performance falls below aspirations. J. General Manage. 44, 73–86. 10.1177/0306307018798143

[B118] Wennberg K. Holmquist C. (2008). Problemistic search and international entrepreneurship. Eur. Manag. J. 26, 441–454. 10.1016/j.emj.2008.09.007

[B119] Wiersema M. F. (1992). Strategic consequences of executive succession within diversified firms. J. Manag. Stud. 29, 73–94. 10.1111/j.1467-6486.1992.tb00653.x

[B120] Wiseman R. M. Gomez-Mejia L. R. (1998). A behavioral agency model of managerial risk taking. Acad. Manage. Rev. 23, 133–153. 10.2307/259103

[B121] Zyung J. D. Shi W. (2022). In retrospect: the influence of chief executive officers' historical relative pay on overconfidence. Strat. Organiz. 20, 627–651. 10.1177/14761270211004891

