# Supplementary material for: Unfinished business: integrating individual decision-makers' experience and incentives to organizational performance feedback theory
Source: Front Psychol. 2023 Jul 5;14:1166185. doi: 10.3389/fpsyg.2023.1166185 (PMC10354647; doi:10.3389/fpsyg.2023.1166185)
Supplement: Supplementary file 1 [file Table_1.docx]

Supplementary Material

Unfinished Business: Integrating Individual Decision-Makers’ Experience and Incentives to Organizational Performance Feedback Theory

Daniela Blettner^1^, Serhan Kotiloglu^2*^, Thomas Lecher^3^

^1^Beedie School of Business, Simon Fraser University, Burnaby, BC, Canada

^2^College of Business Administration, California State University San Marcos, San Marcos, CA, USA

^3^School of Business, Stevens Institute of Technology, Hoboken, NJ, USA

*** Correspondence:**Daniela Blettner
daniela_blettner@sfu.ca

**APPENDIX**: Studies included in the meta-analysis

| **Study** | **Sample Size** | **Data Collection Period** | **Inclusion of Individual-Level Variables** |
| --- | --- | --- | --- |
| Ahn et al., 2020 | 447 observations | 2011-2017 | No |
| Alessandri, 2006 | 2,230 observations | 1998-2003 | Yes |
| Alessandri, 2008 | 128 observations | 1998-2000 | Yes |
| Alexy et al., 2016 | 313 observations | 2008-2010 | No |
| Arora et al., 2011 | 1,522 observations | 2001-2006 | Yes |
| Arrfelt et al., 2012 | 8,266 observations | 1998-2006 | Yes |
| Askin and Bothner, 2016 | 1,019 observations | 2005-2012 | No |
| Audia and Brion, 2007 | 88 observations | 1977-1999 | No |
| Audia and Greve, 2006 | 178 observations | 1974-1995 | No |
| Audia et al., 2000 | 150 observations | 1974-1985 | Yes |
| Barreto, 2012 | 25,452 observations | 1991-1994 | No |
| Baum and Dahlin, 2007 | 189 observations | 1975-2001 | No |
| Baum et al., 2005 | 2,465 observations | 1952-1990 | Yes |
| Ben-Oz and Greve, 2015 | 252 observations | 2007 | Yes |
| Blageova et al., 2020 | 1,887 observations | 2002-2014 | Yes |
| Blettner and Di Lorenzo, 2017 | 1,209 observations | 1990-2003 | No |
| Blettner et al., 2019 | 5,549 observations | 1998-2014 | No |
| Borgholthaus et al., 2021 | 3,032 observations | 2008-2017 | Yes |
| Boyle and Shapira, 2012 | 112 observations | 1990-1999 | Yes |
| Bromiley and Washburn, 2011 | 22,189 observations | 1977-1997 | No |
| Busenbark et al., 2018 | 9,401 observations |  | No |
| Buyl and Boone, 2014 | 298 observations | 2000-2009 | Yes |
| Buyl et al., 2019 | 920 observations | 2006-2008 | Yes |
| Cabral et al., 2023 | 94,876 observations | 1990-2015 | No |
| Calabrò et al., 2018 | 432 observations | 2000-2014 | Yes |
| Castellanata et al., 2015 | 7,223 observations | 1973-2008 | No |
| Ceci et al., 2012 | 2,265 observations | 2001-2006 | No |
| Ceci et al., 2016 | 762 observations | 2001-2006 | No |
| Chen, 2008 | 15,171 observations | 1980-2001 | No |
| Chen and Li, 2021 | 711 observations | 2008-2015 | No |
| Chen et al., 2021 | 3,790 observations | 2007-2020 | Yes |
| Chen and Miller, 2007 | 35,970 observations | 1980-2001 | No |
| Chen and Song, 2020 | 6,543 observations | 2004-2011 | No |
| Chen et al., 2023 | 6,981 observations | 2008-2020 | Yes |
| Cheng et al., 2021 | 7,460 observations | 2011-2017 | Yes |
| Cheon, 2020 | 592 observations |  | No |
| Choi et al., 2019 | 9,566 observations | 1992-2005 | Yes |
| Chrisman and Patel, 2012 | 8,473 observations | 1998-2007 | Yes |
| Clough and Piezunka, 2020 | 432 observations | 1981-2013 | No |
| Dasgupta, 2022 | 24,390 observations | 2010-2019 | No |
| Deb et al., 2019 | 27,984 observations | 1994-2013 | No |
| Delmar and Wennberg, 2007 | 79,587 observations | 1995-2002 | No |
| Deng and Long, 2019 | 10,280 observations | 2011-2016 | Yes |
| Deng et al., 2022 | 7,792 observations | 1998-2012 | No |
| Deperi et al., 2022 | 31,108 observations | 1990-2018 | No |
| Desai, 2008 | 466 observations | 1978-2003 | No |
| Desai, 2013 | 6,632 observations | 1999-2004 | No |
| Desai, 2015a | 607 observations | 2003-2010 | Yes |
| Desai, 2015b | 315 observations | 2003-2010 | No |
| Dong et al., 2021 | 7,237 observations | 2001-2005 | No |
| Dong et al., 2022 | 6,539 observations | 2008-2017 | Yes |
| Duke et al., 2022 | 1,439 observations | 2010-2017 | No |
| Eggers and Kaul, 2018 | 785,490 observations | 1980-1997 | No |
| Ertug and Caner, 2015 | 2,043 observations | 1989-2005 | No |
| Gaba et al., 2012 | 1,424 observations | 1992-2003 | No |
| Gaba et al., 2022 | 5,394 observations | 1999-2016 | Yes |
| Garcia-Garcia et al., 2022 | 852 observations | 1986-2010 | Yes |
| Gao et al., 2021 | 8,273 observations | 2004-2015 | Yes |
| Gao et al., 2022 | 5,163 observations | 2007-2013 | No |
| Gentry and Shen, 2013 | 14,729 observations | 1979-2005 | No |
| Gomez-Meija et al., 2018 | 8,485 observations | 1997-2001 | Yes |
| Goyal and Goyal, 2021 | 2,691 observations | 2010-2017 | No |
| Greve, 1998 | 16,294 observations | 1984-1992 | No |
| Greve, 2003a | 1,672 observations | 1971-1996 | No |
| Greve, 2003b | 185 observations | 1964-1995 | No |
| Greve, 2007 | 258 observations | 1971-2000 | No |
| Greve, 2008 | 4,842 observations | 1911-1996 | No |
| Greve, 2010 | 2,151 observations | 1992-2004 | No |
| Grohsjean et al., 2011 | 493 observations | 1990-2003 | No |
| Grond, 2018 | 860 observations | 2003-2013 | Yes |
| Gubbi et al., 2015 | 2,729 observations | 1992-2005 | No |
| Guo and Peng, 2017 | 16,057 observations | 1988-2015 | No |
| Guo et al., 2019 | 6,814 observations | 2011-2015 | Yes |
| Han, 2023 | 166,682 observations | 2007-2019 | No |
| Harris and Bromiley, 2007 | 868 observations | 1997-2002 | Yes |
| He et al., 2022 | 2,760 observations | 2009-2017 | Yes |
| Hendron et al., 2013 | 494 observations | 1975-1986 | No |
| Hoang and Ener, 2015 | 369 observations | 1979-2000 | No |
| Hu et al., 2017 | 11,657 observations | 1972-2010 | No |
| Hu et al., 2021 | 20,374 observations | 1992-2018 | Yes |
| Hu et al., 2022 | 2,548 observations | 1997-2017 | No |
| Huang et al., 2021a | 3,285 observations | 2009-2017 | Yes |
| Huang et al., 2021b | 136 observations | 2011-2015 | Yes |
| Hui et al., 2021 | 11,190 observations | 1997-2016 | No |
| Iglesias et al., 2019 | 40,224 observations | 1998-2008 | No |
| Iyer and Miller, 2008 | 51,567 observations | 1980-2000 | No |
| Jiang, 2018 | 327,942 observations | 1976-2002 | No |
| Jirasek, 2016 | 199 observations | 2002-2014 | No |
| Jung and Bansal, 2009 | 4,206 observations | 1992-1998 | No |
| Jung et al., 2022 | 6,672 observations |  | No |
| Kacperczyk et al., 2015 | 3,225 observations | 1980-2006 | No |
| Kavadis and Castener, 2015 | 414 observations | 2000-2007 | Yes |
| Kavusan and Frankort, 2019 | 1,016 observations | 1985-2000 | No |
| Ketchen and Palmer, 1999 | 66 observations | 1986-1990 | No |
| Kim and Kim, 2020 | 362 observations | 1995-2016 | Yes |
| Kim and Rhee, 2014 | 331 observations | 1986-2006 | Yes |
| Kim and Rhee, 2017 | 4,163 observations | 1990-2014 | No |
| Kim and Tsai, 2012 | 650 observations | 2004-2006 | No |
| Kim et al., 2015 | 8,799 observations | 1988-2005 | No |
| Kolev and McNamara, 2020 | 3,265 observations | 1999-2014 | Yes |
| Kostopoulos, 2022 | 1,139,961 observations | 2016-2017 | Yes |
| Kotiloglu, 2023 | 1,050 observations | 2012-2020 | Yes |
| Kotiloglu et al., 2018 | 24,902 observations | 2007-2016 | No |
| Kotlar et al., 2013 | 4,903 observations | 2000-2006 | No |
| Kotlar et al., 2014a | 1,019 observations | 2000-2006 | No |
| Kotlar et al., 2014b | 995 observations | 2000-2006 | No |
| Kuusela et al., 2017 | 4,660 observations | 1992-2014 | No |
| Labianca et al., 2009 | 131 observations | 2004 | No |
| Lant and Hewlin, 2002 | 116 observations |  | Yes |
| Lant and Hurley, 1999 | 417 observations |  | Yes |
| Le and Lee, 2021 | 3,133 observations | 2010-2018 | Yes |
| Lee et al., 2020 | 272 observations | 1993-2008 | No |
| Lehman and Hahn, 2013 | 22,603 observations | 2000-2005 | No |
| Lewellyn and Bao, 2015 | 83 observations | 2002-2010 | No |
| Lim, 2015 | 2,004 observations | 1994-2006 | Yes |
| Lim, 2017 | 3,008 observations | 1994-2006 | Yes |
| Lim, 2018 | 2,878 observations | 1994-2006 | Yes |
| Lim and McCann, 2014 | 3,892 observations | 1992-2006 | Yes |
| Lin, 2014 | 5,904 observations | 2000-2008 | No |
| Lin et al., 2012 | 7,290 observations | 1997-2006 | No |
| Lohrke et al., 2006 | 808 observations |  | No |
| Lu and Wong, 2019 | 2,436 observations | 1998-2012 | No |
| Lu et al., 2013 | 4,682 observations | 1999-2008 | No |
| Lucas et al., 2018 | 1,750 observations | 2009 | No |
| Luger, 2023 | 581 observations | 2016-2019 | No |
| Lungeanu et al., 2016 | 392 observations | 1992-2006 | Yes |
| Lv et al., 2019 | 10,170 observations | 2007-2017 | No |
| Lv et al., 2021 | 9,449 observations | 2008-2018 | No |
| Lycosa, 2018 | 3,586 observations | 2014-2015 | No |
| Ma, 2016 | 345 observations | 2000-2012 | Yes |
| Madidan et al., 2022 | 31,676 observations | 2016-2019 | No |
| Madsen, 2013 | 915 observations | 1990-2007 | No |
| Makarevich, 2018 | 9,837 observations | 1970-2003 | No |
| Malen and Vaaler, 2017 | 42,087 observations | 1991-2005 | No |
| Mammen, 2021 | 11,170 observations |  | No |
| Manzaneque et al., 2018 | 3,116 observations | 2001-2013 | No |
| Massini et al., 2015 | 122 observations | 1992-1996 | No |
| Mazzelli et al., 2019 | 9,954 observations | 1998-2012 | No |
| Mezias et al., 2002 | 860 observations | 1995-1997 | Yes |
| Miller, 1994 | 497 observations | 1990-1991 | No |
| Mishina et al., 2010 | 1,749 observations | 1990-1999 | No |
| Moliterno et al., 2007 | 374 observations | 1969-1983 | No |
| Moliterno et al., 2015 | 6,534 observations | 1992-2004 | No |
| Mount and Baer, 2021 | 14,187 observations | 2000-2015 | Yes |
| Murphy et al., 2001 | 810 observations | 1995-1998 | Yes |
| Nagel, 2021 | 985 observations | 2005-2016 | No |
| Nevmerzhytskyi, 2021 | 1,246 observations | 2006-2016 | No |
| O'Brien and David, 2014 | 18,283 observations | 1992-2004 | No |
| Oehler et al., 2019 | 184 observations |  | No |
| Ok and Ahn, 2019 | 325 observations | 2010-2013 | No |
| Park, 2007 | 236,237 observations | 1985-2000 | No |
| Parker et al., 2017 | 1,332 observations | 2006-2009 | No |
| Ploeg et al., 2022 | 51,567 observations | 2010-2017 | No |
| Ref and Shapira, 2016 | 3,926 observations | 1999-2007 | No |
| Rhee et al., 2019 | 1,274 observations | 2001-2008 | No |
| Roman et al., 2022 | 447 observations | 1992-2019 | Yes |
| Rostami, 2008 | 948 observations | 1991-2002 | No |
| Rowley et al., 2017 | 2,736 observations | 2001-2010 | No |
| Ruckman and Blettner, 2020 | 143 observations | 2002-2011 | No |
| Rudy and Johnson, 2016 | 2,190 observations | 1980-2006 | No |
| Ruth et al., 2013 | 29,497 observations | 1980-2000 | No |
| Salge, 2010 | 770 observations | 2002-2007 | No |
| Salge et al., 2015 | 612 observations | 2002-2007 | No |
| Saraf et al., 2018 | 7,330 observations | 1999-2006 | No |
| Saraf et al., 2021 | 7,535 observations | 1999-2006 | No |
| Say and Vasudeva, 2020 | 46,182 observations | 2005-2016 | Yes |
| Schimmer and Brauer, 2012 | 10,719 observations | 1999-2008 | No |
| Schumacher et al., 2020 | 5,482 observations | 1992-2014 | Yes |
| Schwab, 2007 | 70 observations | 1923-1940 | No |
| Sengul and Obloj, 2017 | 1,206 observations | 1998-2004 | No |
| Shi et al., 2020 | 17,727 observations | 2002-2013 | Yes |
| Shijaku et al., 2020 | 7,760 observations | 2002-2013 | No |
| Shimizu, 2007 | 1,544 observations | 1988-1998 | Yes |
| Shipilov et al., 2011 | 21,706 observations | 1979-2001 | No |
| Shou et al., 2020 | 746 observations |  | No |
| Singh and Guha, 2018 | 160 observations | 2007-2010 | No |
| Smulovitz et al., 2020 | 73,401 observations | 2005-2013 | No |
| Sobrepere i Profito et al., 2022 | 23,895 observations | 2009-2016 | Yes |
| Souder and Bromiley, 2012 | 8,241 observations | 1991-2007 | No |
| Su and Si, 2015 | 609 observations | 1995-2010 | No |
| Su and Su, 2017 | 6,539 observations |  | No |
| Sun and Qui, 2022 | 621 observations | 2013-2017 | Yes |
| Tarakci et al., 2016 | 21 observations |  | Yes |
| Titus et al., 2019 | 2,054 observations | 1996-2007 | Yes |
| Tuggle et al., 2010 | 979 observations | 1994-2000 | Yes |
| Tyler and Caner, 2015 | 622 observations | 1997-2007 | No |
| Vidal and Mitchell, 2015 | 504 observations | 1999-2009 | No |
| Villagrasa et al., 2018 | 137 observations | 2006-2008 | Yes |
| Wan et al., 2022 | 9,138 observations | 2011-2019 | Yes |
| Wang and Zhang, 2021 | 487 observations | 2012-2016 | Yes |
| Wang et al., 2021 | 12,327 observations | 2001-2017 | No |
| Wangrow et al., 2019 | 99 observations |  | Yes |
| Wennberg and Holmquist, 2008 | 2,355 observations | 1992-1995 | Yes |
| Wennberg et al., 2016 | 39,890 observations | 1995-2002 | No |
| Xie et al., 2019 | 3,575 observations | 2007-2013 | No |
| Xu and Zeng, 2020 | 1,416 observations | 2008-2013 | No |
| Xu et al., 2019 | 9,633 observations | 2007-2013 | Yes |
| Xue et al., 2022 | 19,876 observations | 2010-2018 | Yes |
| Yang et al., 2017 | 112 observations | 2000-2005 | No |
| Ye et al., 2020 | 56,716 observations | 1974-2018 | No |
| Yu et al., 2018 | 10,618 observations | 1980-2014 | No |
| Zhang, 2018 | 57,302 observations | 2000-2015 | No |
| Zhang and Greve, 2018 | 28,847 observations | 2000-2012 | No |
| Zhong et al., 2021 | 4,386 observations | 2010-2018 | Yes |
| Zhong et al., 2022 | 5,421 observations | 2005-2018 | Yes |

**REFERENCES**

Ahn, S., Cho, C.K., and Cho, T.S. (2020). Performance feedback and organizational learning: the role of regulatory focus. *Management Decision* 59(3)**,** 616-637. doi: 10.1108/md-09-2019-1319.

Alessandri, T.M. (2008). Risk and procedural rationality: a behavioral theory perspective. *Journal of Strategy and Management* 1(2)**,** 198-217. doi: 10.1108/17554250810926375.

Alessandri, T.M., and Khan, R.H. (2006). Market performance and deviance from industry norms: (Mis)alignment of organizational risk and industry risk. *Journal of Business Research* 59(10-11)**,** 1105-1115. doi: 10.1016/j.jbusres.2006.07.004.

Alexy, O., Bascavusoglu-Moreau, E., and Salter, A. (2016). Toward an aspiration-level theory of open innovation. *Industrial and Corporate Change* 25(2)**,** 289-306.

Arora, P., and Dharwadkar, R. (2011). Corporate governance and corporate social responsibility (CSR): The moderating roles of attainment discrepancy and organization slack. *Corporate Governance: An International Review* 19(2)**,** 136-152. doi: 10.1111/j.1467-8683.2010.00843.x.

Arrfelt, M., Wiseman, R.M., and Hult, G.T.M. (2012). Looking backward instead of forward: Aspiration-driven influences on the efficiency of the capital allocation process. *Academy of Management Journal* 56(4)**,** 1081-1103. doi: 10.5465/amj.2010.0879.

Askin, N., and Bothner, M.S. (2016). Status-Aspirational pricing: The "Chivas Regal" strategy in U.S. higher education, 2006-2012. *Administrative Science Quarterly* 61(2)**,** 217-253. doi: 10.1177/0001839216629671.

Audia, P.G., and Brion, S. (2007). Reluctant to change: Self-enhancing responses to diverging performance measures. *Organizational Behavior and Human Decision Processes* 102(2)**,** 255-269. doi: 10.1016/j.obhdp.2006.01.007.

Audia, P.G., and Greve, H.R. (2006). Less likely to fail: Low performance, firm size, and factory expansion in the shipbuilding industry. *Management Science* 52(1)**,** 83-94. doi: 10.1287/mnsc.1050.0446.

Audia, P.G., Locke, E.A., and Smith, K.G. (2000). The paradox of success: An archival and a laboratory study of strategic persistence following radical environmental change. *Academy of Management Journal* 43(5)**,** 837-853.

Barreto, I. (2012). A behavioral theory of market expansion based on the opportunity prospects rule. *Organization Science* 23(4)**,** 1008-1023. doi: 10.1287/orsc.1110.0674.

Baum, J.A., and Dahlin, K.B. (2007). Aspiration performance and railroads’ patterns of learning from train wrecks and crashes. *Organization Science* 18(3)**,** 368-385. doi: 10.1287/orsc.1060.0239.

Baum, J.A., Rowley, T.J., Shipilov, A.V., and Chuang, Y.-T. (2005). Dancing with strangers: Aspiration performance and the search for underwriting syndicate partners. *Administrative Science Quarterly* 50(4)**,** 536-575.

Ben-Oz, C., and Greve, H.R. (2015). Short- and long-term performance feedback and absorptive capacity. *Journal of Management* 41(7)**,** 1827-1853. doi: 10.1177/0149206312466148.

Blagoeva, R.R., Mom, T.J., Jansen, J.J., and George, G. (2020). Problem-solving or self-enhancement? A power perspective on how CEOs affect R&D search in the face of inconsistent feedback. *Academy of Management Journal* 63(2)**,** 332-355.

Blettner, D., Atanasova, C., Lucas, G.J.M., and Gedajlovic, E.R. (2019). How does financial performance feedback and personal reputation affect timing of future projects? *Academy of Management Proceedings* 2019(1)**,** 12249.

Blettner, D., and Di Lorenzo, F. (2017). "Multiple performances, aspiration levels and partnering behavior: The case of the pharmaceutical industry". (Available at SSRN).

Borgholthaus, C.J., Iyer, D.N., and O'Brien, J.P. (2021). The effects of firm aspirational performance on changes in leadership structure. *Journal of Business Research* 129**,** 319-327. doi: 10.1016/j.jbusres.2021.03.009.

Boyle, E., and Shapira, Z. (2012). The liability of leading: Battling aspiration and survival goals in the Jeopardy! Tournament of Champions. *Organization Science* 23(4)**,** 1100-1113. doi: 10.1287/orsc.1110.0690.

Bromiley, P., and Washburn, M. (2011). Cost reduction vs innovative search in R&D. *Journal of Strategy and Management* 4(3)**,** 196-214. doi: 10.1108/17554251111152243.

Busenbark, J.R., Arrfelt, M., Semadeni, M., and Withers, M.C. (2018). The role of firm performance aspirations in managers' internal capital allocation strategies. . *Academy of Management Proceedings* 2018(1)**,** 10510.

Buyl, T., and Boone, C. (2014). "Exploration versus exploitation: The differential impact of historical and social comparison performance feedback on executives' cognitive orientation," in *Behavioral Strategy: Emerging Perspectives*. Information Age Publishing - IAP), 27-54.

Cabral, J.J., Iyer, D.N., and O’Brien, J.P. (2022). EXPRESS: How the Ghosts of Past Experience Haunt Problemistic Search. *Strategic Organization* in press.

Calabrò, A., Minichilli, A., Amore, M.D., and Brogi, M. (2018). The courage to choose! Primogeniture and leadership succession in family firms. *Strategic Management Journal* 39(7)**,** 2014-2035. doi: 10.1002/smj.2760.

Castellaneta, F., Gottschalg, O., Kacperczyk, A., and Wright, M. (2015). The disadvantage of incomplete performance feedback: Evidence from private equity–backed buyouts. *Working Paper*. doi: 10.13140/RG.2.1.4903.4962.

Ceci, F., Masciarelli, F., and Prencipe, A. (Year). "Mind the gap: Adapting and reorienting organizational architectures", in: *DRUID*).

Ceci, F., Masciarelli, F., and Prencipe, A. (2016). Changes in organizational architecture: Aspiration levels, performance gaps and organizational change. *International Journal of Innovation and Technology Management* 13(01). doi: 10.1142/s0219877016500024.

Chen, W., Zhong, X., and Lan, H. (2023). Innovation for survival: The scope of negative attainment discrepancy and enterprise R&D investment. *Industrial Marketing Management* 108**,** 190-204. doi: 10.1016/j.indmarman.2022.12.002.

Chen, W.-R. (2008). Determinants of firms' backward- and forward-looking R&D search behavior. *Organization Science* 19(4)**,** 609-622. doi: 10.1287/orsc.1070.0320.

Chen, W.-R., and Miller, K.D. (2007). Situational and institutional determinants of firms' R&D search intensity. *Strategic Management Journal* 28(4)**,** 369-381. doi: 10.1002/smj.594.

Chen, X., and Li, Q. (2021). Environmental regulation, subsidy and underperforming firms' R&D expenditure: evidence from Chinese listed companies. *International Journal of Technology Management* 85(2-4).

Chen, Y., and Song, M. (2020). The persistence and dynamics of new venture growth. *Small Business Economics*. doi: 10.1007/s11187-020-00411-2.

Cheng, L., Nan, M., Fang, A.J., and Xie, E. (2021). "Performance feedback and firms’ relative strategic emphasis", in: *2021 Academy of Management Annual Meeting.* (Virtual).

Cheon, O. (2020). How do performance gaps shape managerial strategy? The role of sector-differences in U.S. nursing homes. *International Public Management Journal***,** 1-19. doi: 10.1080/10967494.2020.1815917.

Choi, J., Rhee, M., and Kim, Y.-C. (2019). Performance feedback and problemistic search: The moderating effects of managerial and board outsiderness. *Journal of Business Research* 102**,** 21-33. doi: 10.1016/j.jbusres.2019.04.039.

Chrisman, J.J., and Patel, P.C. (2012). Variations in R&D investments of family and nonfamily firms: Behavioral agency and myopic loss aversion perspectives. *Academy of Management Journal* 55(4)**,** 976-997. doi: 10.5465/ami.2011.0211.

Clough, D.R., and Piezunka, H. (2020). Tie Dissolution in Market Networks: A Theory of Vicarious Performance Feedback. *Administrative Science Quarterly* 65(4)**,** 972-1017. doi: 10.1177/0001839219899606.

DasGupta, R. (2022). Financial performance shortfall, ESG controversies, and ESG performance: Evidence from firms around the world. *Finance Research Letters* 46. doi: 10.1016/j.frl.2021.102487.

Deb, P., David, P., O'Brien, J.P., and Duru, A. (2019). Attainment discrepancy and investment: Effects on firm performance. *Journal of Business Research* 99**,** 186-196. doi: 10.1016/j.jbusres.2019.02.047.

Delmar, F., and Wennberg, K. (2007). Risk taking, aspiration levels and the evolution of new ventures. *Frontiers of Entrepreneurship Research* 27(13).

Deng, X., and Long, X. (2019). Financial Performance Gaps and Corporate Social Responsibility. *Sustainability* 11(12). doi: 10.3390/su11123438.

Deng, Z., Li, T., and Liesch, P.W. (2022). Performance shortfalls and outward foreign direct investment by MNE subsidiaries: Evidence from China. *International Business Review* 31(3). doi: 10.1016/j.ibusrev.2021.101952.

Deperi, J., Bertrand, O., Meschi, P.-X., and Nesta, L. (2022). An organizational learning approach to digital and non-digital firm acquisition behavior. *European Management Journal* 40(6)**,** 873-882. doi: 10.1016/j.emj.2022.09.005.

Desai, V.M. (2008). Constrained growth: How experience, legitimacy, and age influence risk taking in organizations. *Organization Science* 19(4)**,** 594-608. doi: 10.1287/orsc.1070.0335.

Desai, V.M. (2013). Learning to behave badly: performance feedback and illegal organizational action. *Industrial and Corporate Change* 23(5)**,** 1327-1355. doi: 10.1093/icc/dtt043.

Desai, V.M. (2015a). The behavioral theory of the (governed) firm: Corporate board bnfluences on organizations' responses to performance shortfalls. *Academy of Management Journal*.

Desai, V.M. (2015b). Learning through the distribution of failures within an organization: Evidence from heart bypass surgery performance. *Academy of Management Journal* 58(4)**,** 1032-1050. doi: 10.5465/amj.2013.0949.

Dong, J.Q., Karhade, P.P., Rai, A., and Xu, S.X. (2021). How Firms Make Information Technology Investment Decisions: Toward a Behavioral Agency Theory. *Journal of Management Information Systems* 38(1)**,** 29-58. doi: 10.1080/07421222.2021.1870382.

Dong, M., Wang, L., Yang, D., and Zhou, K.Z. (2022). Performance feedback and export intensity of Chinese private firms: Moderating roles of institution-related factors. *International Business Review* 31(3). doi: 10.1016/j.ibusrev.2021.101948.

Duke, J., Havakhor, T., Mui, R., and Parker, O. (2021). How Starting Strategy and Network Structure Shape Problemistic Search: An Examination of Venture Capital Firms. *Entrepreneurship Theory and Practice* 46(5)**,** 1344-1373. doi: 10.1177/10422587211033574.

Eggers, J.P., and Kaul, A. (2018). Motivation and ability? A behavioral perspective on the pursuit of radical invention in multi-technology incumbents. *Academy of Management Journal* 61(1)**,** 67-93. doi: 10.5465/amj.2015.1123.

Ertug, G., and Castellucci, F. (2015). Who shall get more? How intangible assets and aspiration levels affect the valuation of resource providers. *Strategic Organization* 13(1)**,** 6-31. doi: 10.1177/1476127014561019.

Gaba, V., and Bhattacharya, S. (2012). Aspirations, innovation, and corporate venture capital: A behavioral perspective. *Strategic Entrepreneurship Journal* 6(2)**,** 178-199. doi: 10.1002/sej.1133.

Gaba, V., Lee, S., Meyer-Doyle, P., and Zhao-Ding, A. (2022). Prior Experience of Managers and Maladaptive Responses to Performance Feedback: Evidence from Mutual Funds. *Organization Science*. doi: 10.1287/orsc.2022.1605.

Gao, R., Lu, J.W., Hu, H.W., and Martin, G. (2023). A tale of two distractions: How institutional forces influence

R&D

‐based problemistic search in transition economies? *Journal of Product Innovation Management*. doi: 10.1111/jpim.12657.

Gao, Y., Yang, H., and Zhang, M. (2021). Too bad to fear, too good to dare? Performance feedback and corporate misconduct. *Journal of Business Research* 131**,** 1-11. doi: 10.1016/j.jbusres.2021.03.041.

García-García, R., García-Canal, E., and Guillén, M.F. (2022). Walking on thin ice: CEOs′ internationalization decisions in underperforming firms. *Long Range Planning* 55(5). doi: 10.1016/j.lrp.2022.102243.

Gentry, R.J., and Shen, W. (2013). The impacts of performance relative to analyst forecasts and analyst coverage on firm R&D intensity. *Strategic Management Journal* 34(1)**,** 121-130. doi: 10.1002/smj.1997.

Gomez-Mejia, L.R., Patel, P.C., and Zellweger, T.M. (2018). In the horns of the dilemma: Socioemotional wealth, financial wealth, and acquisitions in family firms. *Journal of Management* 44(4)**,** 1369-1397. doi: 10.1177/0149206315614375.

Goyal, L., and Goyal, V. (2021). Performance shortfall, feedback interpretation and R&D search: The differential effects of peers’ performance below historical and social aspirations. *British Journal of Management*. doi: 10.1111/1467-8551.12532.

Greve, H.R. (1998). Performance, aspirations, and risky organizational change. *Administrative Science Quarterly* 43(1)**,** 58-86.

Greve, H.R. (2003a). A behavioral theory of R&D expenditures and innovations: Evidence from shipbuilding. *Academy of Management Journal* 46(6)**,** 685-702.

Greve, H.R. (2003b). Investment and the behavioral theory of the firm: Evidence from shipbuilding. *Industrial and Corporate Change* 12(5)**,** 1051-1076.

Greve, H.R. (2007). Exploration and exploitation in product innovation. *Industrial and Corporate Change* 16(5)**,** 945-975. doi: 10.1093/icc/dtm013.

Greve, H.R. (2008). A behavioral theory of firm growth: Sequential attention to size and performance goals. *Academy of Management Journal* 51(3)**,** 476-494.

Greve, H.R. (2010). Positional rigidity: low performance and resource acquisition in large and small firms. *Strategic Management Journal* 32(1)**,** 103-114. doi: 10.1002/smj.875.

Grohsjean, T., Kretschmer, T., and Stieglitz, N. (2011). Performance feedback, firm resources, and strategic change. *DRUID Working Paper* 11-02.

Grond, A. A. (2023). Organizations’ Response to CSR Performance Feedback: The Role of Financial Performance Feedback and CEO-Board Interlocks. *Tilburg University Working Paper.*

Gubbi, S.R., Aulakh, P.S., and Ray, S. (2015). International search behavior of business group affiliated firms: Scope of institutional changes and intragroup heterogeneity. *Organization Science* 26(5)**,** 1485-1501. doi: 10.1287/orsc.2015.0990.

Guo, B., and Ding, P. (2017). Invention or incremental improvement? Simulation modeling and empirical testing of firm patenting behavior under performance aspiration. *Decision Support Systems* 102**,** 32-41. doi: 10.1016/j.dss.2017.07.001.

Guo, B., and Ding, P. (2019). A behavioral theory of patent application rhythm. *Management Decision* 58(4)**,** 743-758. doi: 10.1108/md-11-2018-1271.

Han, S. (2022). The effect of performance feedback on strategic alliance formation and R&D intensity. *European Management Journal*. doi: 10.1016/j.emj.2022.03.010.

Harris, J., and Bromiley, P. (2007). Incentives to cheat: The influence of executive compensation and firm performance on financial misrepresentation. *Organization Science* 18(3)**,** 350-367. doi: 10.1287/orsc.1060.0241.

He, L., Huang, L., and Yang, G. (2021). Invest in Innovation or Not? How Managerial Cognition and Attention Allocation Shape Corporate Responses to Performance Shortfalls. *Management and Organization Review* 17(4)**,** 815-850. doi: 10.1017/mor.2021.58.

Hendron, M.G., Bednar, M.K., and Henderson, A.D. (2013). How performance relative to aspiration levels affects strategic responses to technological discontinuity. *Working Paper*.

Hoang, H., and Ener, H. (2015). Unpacking experience effects in developing novel products for new markets. *Strategic Organization* 13(4)**,** 261-283. doi: 10.1177/1476127015590141.

Hu, S., Gentry, R.J., Quigley, T.J., and Boivie, S. (2021). Who's in the Driver's Seat? Exploring Firm-Level vs. CEO-Level Effects on Problemistic Search. *Journal of Management*. doi: 10.1177/01492063211063823.

Hu, S., Gu, Q., and Xia, J. (2022). Problemistic Search of the Embedded Firm: The Joint Effects of Performance Feedback and Network Positions on Venture Capital Firms’ Risk Taking. *Organization Science* 33(5)**,** 1889-1908. doi: 10.1287/orsc.2021.1513.

Hu, S., He, Z.-L., Blettner, D.P., and Bettis, R.A. (2017). Conflict inside and outside: Social comparisons and attention shifts in multidivisional firms. *Strategic Management Journal* 38(7)**,** 1435-1454. doi: 10.1002/smj.2556.

Huang, L., He, L., and Yang, G. (2021a). Performance Shortfalls and R&D Investment Change: Aspirations, Actions, and Expectations. *Sustainability* 13(6). doi: 10.3390/su13063006.

Huang, Y.-C., Chin, Y.-C., and Lee, C.-Y. (2021b). Which executive characteristics influence risk-taking behaviours: evidence from Taiwanese companies. *Asia Pacific Business Review***,** 1-27. doi: 10.1080/13602381.2021.1917159.

Hui, K.N.-C., Gong, Y., Cui, Q., and Jiang, N. (2021). Foreign investment or divestment as a near-term solution to performance shortfalls? The moderating role of vicarious learning. *Asia Pacific Journal of Management*. doi: 10.1007/s10490-021-09778-6.

Iglesias, A.E., and Bogner, W.C. (2019). Do competitors always matter in exit decisions? A behavioral perspective. *International Journal of Business* 24(2)**,** 132-150.

Iyer, D.N., and Miller, K.D. (2008). Performance feedback, slack, and the timing of acquisitions. *Academy of Management Journal* 51(4)**,** 808-822.

Jiang, G.F., and Holburn, G.L.F. (2018). Organizational performance feedback effects and international expansion. *Journal of Business Research* 90**,** 48-58. doi: 10.1016/j.jbusres.2018.04.034.

Jirásek, M. (2023). "Innovative behavior of US pharmaceutical firms", in: *Proceedings of the 4th International Conference on Innovation and Entrepreneurship*).

Jung, J.C., and Bansal, P. (2009). How firm performance affects internationalization. *Management International Review* 49(6)**,** 709-732. doi: 10.1007/s11575-009-0014-7.

Jung, H., Lee, Y. G., & Park, S. H. (2022). Just Diverse Among Themselves: How Does Negative Performance Feedback Affect Boards’ Expertise vs. Ascriptive Diversity?. Organization Science.

Kacperczyk, A., Beckman, C.M., and Moliterno, T.P. (2015). Disentangling risk and change: Internal and external social comparison in the mutual fund industry. *Administrative Science Quarterly* 60(2)**,** 228-262. doi: 10.1177/0001839214566297.

Kavadis, N., and Castañer, X. (2015). Who drives corporate restructuring? Co-Existing owners in French firms. *Corporate Governance: An International Review* 23(5)**,** 417-433. doi: 10.1111/corg.12108.

Kavusan, K., and Frankort, H.T.W. (2019). A behavioral theory of alliance portfolio reconfiguration: Evidence from pharmaceutical biotechnology. *Strategic Management Journal* 40(10)**,** 1668-1702. doi: 10.1002/smj.3041.

Ketchen Jr, D.J., and Palmer, T.B. (1999). Strategic responses to poor organizational performance. *Journal of Management* 25(5)**,** 683-706.

Kim, E., and Rhee, M. (2017a). How airlines learn from airline accidents: An empirical study of how attributed errors and performance feedback affect learning from failure. *Journal of Air Transport Management* 58**,** 135-143. doi: 10.1016/j.jairtraman.2016.10.007.

Kim, J.-Y., Finkelstein, S., and Haleblian, J. (2015). All aspirations are not ceated equal: The differential effects of historical and social aspirations on acquisition behavior. *Academy of Management Journal* 58(5)**,** 1361-1388.

Kim, K.-H., and Tsai, W. (2012). Social comparison among competing firms. *Strategic Management Journal* 33(2)**,** 115-136. doi: 10.1002/smj.945.

Kim, M., and Kim, T. (2020). When Do CEOs Engage in CSR Activities? Performance Feedback, CEO Ownership, and CSR. *Sustainability* 12(19). doi: 10.3390/su12198195.

Kim, T., and Rhee, M. (2017b). Structural and behavioral antecedents of change: Status, distinctiveness, and relative performance. *Journal of Management* 43(3)**,** 716-741. doi: 10.1177/0149206314541150.

Kolev, K.D., and McNamara, G. (2020). The role of top management teams in firm responses to performance shortfalls. *Strategic Organization* (in press).

Kostopoulos, K., Syrigos, E., and Kuusela, P. (2023). Responding to Inconsistent Performance Feedback on Multiple Goals: The Contingency Role of Decision Maker's Status in Introducing Changes. *Long Range Planning* 56(1). doi: 10.1016/j.lrp.2022.102269.

Kotiloglu, S. (2023). Exploring how organizational performance feedback influences corporate social responsibility (CSR): the moderating role of LGBT inclusion. *Journal of Strategy and Management*. doi: 10.1108/jsma-09-2022-0170.

Kotiloglu, S., Lechler, T., and Chen, Y. (2018). A longitudinal analysis on the effects of strategic change on the sustainability of hyper growth. *Academy of Management Proceedings* 2018**,** 12852.

Kotlar, J., De Massis, A., Fang, H., and Frattini, F. (2014a). Strategic reference points in family firms. *Small Business Economics* 43(3)**,** 597-619. doi: 10.1007/s11187-014-9556-6.

Kotlar, J., De Massis, A., Frattini, F., Bianchi, M., and Fang, H. (2013). Technology acquisition in family and nonfamily firms: A longitudinal analysis of Spanish manufacturing firms. *Journal of Product Innovation Management* 30(6)**,** 1073-1088. doi: 10.1111/jpim.12046.

Kotlar, J., Fang, H., De Massis, A., and Frattini, F. (2014b). Profitability goals, control goals, and the R&D investment decisions of family and nonfamily firms. *Journal of Product Innovation Management* 31(6)**,** 1128-1145. doi: 10.1111/jpim.12165.

Kuusela, P., Keil, T., and Maula, M. (2017). Driven by aspirations, but in what direction? Performance shortfalls, slack resources, and resource-consuming vs. resource-freeing organizational change. *Strategic Management Journal* 38(5)**,** 1101-1120. doi: 10.1002/smj.2544.

Labianca, G., Fairbank, J.F., Andrevski, G., and Parzen, M. (2009). Striving toward the future: Aspiration-performance discrepancies and planned organizational change. *Strategic Organization* 7(4)**,** 433-466. doi: 10.1177/1476127009349842.

Lant , T.K., and Hewlin, P.F. (2002). Information cues and decision making: The effects of learning, momentum and socual comparison in competing teams. *Group & Organization Management* 27(3)**,** 374-407.

Lant, T.K., and Hurley, A.E. (1999). A contingency model of response to performance feedback: Escalation of commitment and incremental adaptation in resource investment decisions. *Group & Organization Management* 24(4)**,** 421-437.

Le, Q.-A., and Lee, C.-Y. (2021). Below-aspiration performance and risk-taking behaviour in the context of Taiwanese electronic firms: a contingency analysis. *Asia Pacific Business Review***,** 1-24. doi: 10.1080/13602381.2021.1932091.

Lee, J., Rhee, M., and Park, K.M. (2020). Looking backward through the looking glass: Reference groups and social comparison. *Journal of Management & Organization* 26(1)**,** 110-131. doi: 10.1017/jmo.2018.58.

Lehman, D.W., and Hahn, J. (2013). Momentum and organizational risk taking: Evidence from the National Football League. *Management Science* 59(4)**,** 852-868. doi: 10.1287/mnsc.1120.1574.

Lewellyn, K.B., and Bao, S.R. (2015). R&D investment in the global paper products industry: A behavioral theory of the firm and national culture perspective. *Journal of International Management* 21(1)**,** 1-17. doi: 10.1016/j.intman.2014.12.001.

Lim, E. (2015). The role of reference point in CEO restricted stock and its impact on R&D intensity in high-technology firms. *Strategic Management Journal* 36(6)**,** 872-889. doi: 10.1002/smj.2252.

Lim, E. (2017). CEO option wealth and firm risk-taking: An analysis of multiple reference points. *Long Range Planning* 50(6)**,** 809-825. doi: 10.1016/j.lrp.2016.12.013.

Lim, E. (2018). Social pay reference point, external environment, and risk taking: An integrated behavioral and social psychological view. *Journal of Business Research* 82**,** 68-78. doi: 10.1016/j.jbusres.2017.08.001.

Lim, E., and McCann, B.T. (2014). Performance feedback and firm risk taking: The moderating effects of CEO and outside director stock options. *Organization Science* 25(1)**,** 262-282. doi: 10.1287/orsc.2013.0830.

Lin, S.-H., Chen, Y.-J., Hsu, A.-C., Liu, Y.-C.A., and Wang, H.-C. (2012). Foreign direct investment behavir: Problemistic or slack search. *African Journal of Business Management* 6(28)**,** 8250-8260.

Lin, W.-T. (2014). How do managers decide on internationalization processes? The role of organizational slack and performance feedback. *Journal of World Business* 49(3)**,** 396-408. doi: 10.1016/j.jwb.2013.08.001.

Lohrke, F.T., Kreiser, P.M., and Weaver, K.M. (2006). The influence of current firm performance on future SME alliance formation intentions: A six-country study. *Journal of Business Research* 59(1)**,** 19-27. doi: 10.1016/j.jbusres.2005.02.002.

Lu, L.-H., and Fang, S.-C. (2014). Problematic search, slack search and institutional logic in corporate R&D strategy: An empirical analysis of Taiwanese electronics firms. *Journal of Management & Organization* 19(06)**,** 659-678. doi: 10.1017/jmo.2014.11.

Lu, L.-H., and Wong, P.-K. (2019). Performance feedback, financial slack and the innovation behavior of firms. *Asia Pacific Journal of Management* 36(4)**,** 1079-1109. doi: 10.1007/s10490-018-9634-4.

Lucas, G.J., Knoben, J., and Meeus, M.T. (2018). Contradictory yet coherent? Inconsistency in performance feedback and R&D investment change. *Journal of Management* 44(2)**,** 658–681.

Luger, J. (2023). Who depends on why: Toward an endogenous, purpose‐driven mechanism in organizations' reference selection. *Strategic Management Journal*. doi: 10.1002/smj.3486.

Lungeanu, R., Stern, I., and Zajac, E.J. (2016). When do firms change technology-sourcing vehicles? The role of poor innovative performance and financial slack. *Strategic Management Journal* 37(5)**,** 855-869. doi: 10.1002/smj.2371.

Lv, D.D., Chen, W., Zhu, H., and Lan, H. (2019). How does inconsistent negative performance feedback affect the R&D investments of firms? A study of publicly listed firms. *Journal of Business Research* 102**,** 151-162. doi: 10.1016/j.jbusres.2019.04.045.

Lv, D.D., Zhu, H., Chen, W., and Lan, H. (2021). Negative performance feedback and firm cooperation: How multiple upward social comparisons affect firm cooperative R&D. *Journal of Business Research* 132**,** 872-883. doi: 10.1016/j.jbusres.2020.11.018.

Lyócsa, Š., Výrost, T., and Baumöhl, E. (2018). Social aspirations in European banks: peerinfluenced risk behavior. *ZBW - Deutsche Zentralbibliothek* *für Wirtschaftswissenschaften, Leibniz-Informationszentrum Wirtschaft, Kiel und Hamburg*.

Ma, L. (2016). Performance feedback, government goal-setting and aspiration level adaptation: Evidence from Chinese provinces. *Public Administration* 94(2)**,** 452-471. doi: 10.1111/padm.12225.

Madadian, O., and Van den Broeke, M. (2022). R&D investments in response to performance feedback: moderating effects of firm risk profile and business strategy. *Applied Economics* 55(7)**,** 802-822. doi: 10.1080/00036846.2022.2094879.

Madsen, P.M. (2013). Perils and profits: A reexamination of the link between profitability and safety in U.S. aviation. *Journal of Management* 39(3)**,** 763-791. doi: 10.1177/0149206310396374.

Makarevich, A. (2018). Performance feedback as a cooperation “switch”: A behavioral perspective on the success of venture capital syndicates among competitors. *Strategic Management Journal* 39(12)**,** 3247-3272. doi: 10.1002/smj.2722.

Malen, J., and Vaaler, P.M. (2017). Organizational slack, national institutions and innovation effort around the world. *Journal of World Business* 52(6)**,** 782-797. doi: 10.1016/j.jwb.2017.07.001.

Mammen, J. (2021). How aspiration and expectation shortfalls drive strategic investments. *Problems and Perspectives in Management* 19(1)**,** 470-476. doi: 10.21511/ppm.19(1).2021.39.

Manzaneque, M., Rojo-Ramírez, A.A., Diéguez-Soto, J., and Martínez-Romero, M.J. (2020). How negative aspiration performance gaps affect innovation efficiency. *Small Business Economics* 54(1)**,** 209-233. doi: 10.1007/s11187-018-0091-8.

Massini, S., Lewin, A.Y., and Greve, H.R. (2005). Innovators and imitators: Organizational reference groups and adoption of organizational routines. *Research Policy* 34(10)**,** 1550-1569. doi: 10.1016/j.respol.2005.07.004.

Mazzelli, A., De Massis, A., Petruzzelli, A.M., Del Giudice, M., and Khan, Z. (2020). Behind ambidextrous search: The microfoundations of search in family and non-family firms. *Long Range Planning* 53(6). doi: 10.1016/j.lrp.2019.05.002.

Mezias, S.J., Chen, Y.-R., and Murphy, P.R. (2002). Aspiration-Level adaptation in an American financial services organization: A field study. *Management Science* 48(10)**,** 1285-1300. doi: 10.1287/mnsc.48.10.1285.277.

Miller, K.D. (1994). Diversification responses to environmental uncertainties. *Purdue CIBER Working Papers - 82*.

Mishina, Y., Dykes, B.J., Block, E.S., and Pollock, T.G. (2010). Why “good” firms do bad things: The effects of high aspirations, high expectations, and prominence on the incidence of corporate illegality. *Academy of Management Journal* 53(4)**,** 701-722.

Moliterno, T.P., Beck, N., Beckman, C.M., and Meyer, M. (2015). Knowing your place: Social performance feedback in good times and bad times. *Organization Science* 25(6)**,** 1684-1702. doi: 10.1287/orsc.2014.0923.

Moliterno, T.P., and Wiersema, M.F. (2007). Firm performance, rent appropriation, and the strategic resource divestment capability. *Strategic Management Journal* 28**,** 1065-1087. doi: 10.1002/smj.

Mount, M.P., and Baer, M. (2021). CEOs’ Regulatory Focus and Risk-Taking When Firms Perform Below and Above the Bar. *Journal of Management* (in press).

Murphy, P.R., Mezias , S.J., and Chen, Y.-R. (2001). "Adapting aspirations to feedback: The role of success and failure," in *Organizational Cognition: Computation and Interpretation,* eds. T.K. Lant & Z. Shapira. (Mahwah, NJ: Lawrence Erlbaum Associates).

Nagel, F. (2021). The role of time in organizational aspirations: Past-and future-oriented performance feedback. *Academy of Management Proceedings* 2021(1)**,** 13221.

Nevmerzhytskyi, S. (2021). "The silence of the slacked: The negative side effect of slack on problemistic search". Ivey Business School, Western University).

O'Brien, J.P., and David, P. (2014). Reciprocity and R&D search: Applying the behavioral theory of the firm to a communitarian context. *Strategic Management Journal* 35(4)**,** 550-565. doi: 10.1002/smj.2105.

Oehler, P.J., Stumpf-Wollersheim, J., and Welpe, I.M. (2018). Never change a winning routine? How performance feedback affects routine change. *Industrial and Corporate Change*. doi: 10.1093/icc/dty049.

Ok, C., and Ahn, H.S. (2019). How Does Entrepreneurial Orientation Influence the Sustainable Growth of SMEs? The Role of Relative Performance. *Sustainability* 11(19). doi: 10.3390/su11195178.

Park, K.M. (2007). Antecedents of convergence and divergence in strategic positioning: The effects of performance and aspiration on the direction of strategic change. *Organization Science* 18(3)**,** 386-402. doi: 10.1287/orsc.1060.0240.

Parker, O.N., Krause, R., and Covin, J.G. (2017). Ready, set, slow: How aspiration-relative product quality impacts the rate of new product introduction. *Journal of Management* 43(7)**,** 2333-2356. doi: 10.1177/0149206315569314.

Ploeg, M., Knoben, J., and Vermeulen, P. (2022). We are in it together: Communitarianism and the performance-innovation relationship✰. *Research Policy* 51(5). doi: 10.1016/j.respol.2022.104507.

Ref, O., and Shapira, Z. (2017). Entering new markets: The effect of performance feedback near aspiration and well below and above it. *Strategic Management Journal* 38(7)**,** 1416-1434. doi: 10.1002/smj.2561.

Rhee, L., Ocasio, W., and Kim, T. (2019). Performance feedback in hierarchical business groups: The cross-level effects of cognitive accessibility on R&D search behavior. *Organization Science* 30(1)**,** 51-69.

Roman, A., & Hill, A. (2022). Performance Below Historical Aspirations, Team Size and Member Contribution Dispersion. In Academy of Management Proceedings (Vol. 2022, No. 1, p. 12184). Briarcliff Manor, NY 10510: Academy of Management.

Rostami, M. (2008). "The joint effect of organizational performance and slack resources on corporate acquisitions in the oil industry", in: *Administrative Sciences Association of Canada.* (Halifax, Nova Scotia, Canada).

Rowley, T.J., Shipilov, A.V., and Greve, H.R. (2017). Board reform versus profits: The impact of ratings on the adoption of governance practices. *Strategic Management Journal* 38(4)**,** 815-833. doi: 10.1002/smj.2545.

Ruckman, K., and Blettner, D. (2020). Aspiration adaptation to multiple performance comparisons: Generic strategy as information filter. *Academy of Management Proceedings* 2020(1)**,** 17165.

Rudy, B.C., and Johnson, A.F. (2016). Performance, aspirations, and market versus nonmarket investment. *Journal of Management* 42(4)**,** 936-959. doi: 10.1177/0149206313503017.

Ruth, D., Iyer, D.N., and Sharp, B.M. (2013). Motivation and ability in the decision to acquire. *Journal of Business Research* 66(11)**,** 2287-2293. doi: 10.1016/j.jbusres.2012.02.044.

Salge, T.O. (2010). A behavioral model of innovative search: Evidence from public hospital services. *Journal of Public Administration Research and Theory* 21(1)**,** 181-210. doi: 10.1093/jopart/muq017.

Salge, T.O., Kohli, R., and Barrett, M. (2015). Investing in information systems: On the behavioral and institutional search mechanisms underpinning hospitals' IS investment decisions. *MIS Quarterly* 39(1)**,** 61-89.

Saraf, N., Blettner, D., and Dasgupta, S. (2018). How do you think you are doing? Managerial perceptions, aspirations and innovation. *Academy of Management Proceedings* 2018(1)**,** 17192.

Saraf, N., Dasgupta, S., and Blettner, D. (2021). EXPRESS: How do managerial perceptions of performance feedback affect innovation? *Strategic Organization* (in press).

Say, G., and Vasudeva, G. (2020). Learning from Digital Failures? The Effectiveness of Firms’ Divestiture and Management Turnover Responses to Data Breaches. *Strategy Science* 5(2)**,** 117-142. doi: 10.1287/stsc.2020.0106.

Schimmer, M., and Brauer, M. (2012). Firm performance and aspiration levels as determinants of a firm's strategic repositioning within strategic group structures. *Strategic Organization* 10(4)**,** 406-435. doi: 10.1177/1476127012457983.

Schumacher, C., Keck, S., and Tang, W. (2020). Biased interpretation of performance feedback: The role of CEO overconfidence. *Strategic Management Journal* 41(6)**,** 1139-1165. doi: 10.1002/smj.3138.

Schwab, A. (2007). Incremental organizational learning from multilevel information sources. *Organization Science* 18(2)**,** 233-251. doi: 10.i287/orsc.

Sengul, M., and Obloj, T. (2017). Better safe than sorry: Subsidiary performance feedback and internal governance in multiunit firms. *Journal of Management* 43(8)**,** 2526-2554. doi: 10.1177/0149206316677298.

Shi, W., Chen, G., and Li, B. (2022). Problem Solving or Responsibility Avoidance? The Role of CEO Internal Attribution Tendency in Shaping Corporate Downsizing in Response to Performance Shortfalls. *Journal of Management Studies*. doi: 10.1111/joms.12896.

Shijaku, E., Larraza-Kintana, M., and Urtasun-Alonso, A. (2018). Network centrality and organizational aspirations: A behavioral interaction in the context of international strategic alliances. *Journal of International Business Studies* 51(5)**,** 813-828. doi: 10.1057/s41267-018-0166-4.

Shimizu, K. (2007). Prospect Theory, Behavioral Theory, and the Threat-Rigidity Thesis: Combinative effects on organizational decisions to divest formerly acquired units. *Academy of Management Journal* 50(6)**,** 1495-1514.

Shipilov, A.V., Li, S.X., and Greve, H.R. (2011). The prince and the pauper: Search and brokerage in the initiation of status-heterophilous ties. *Organization Science* 22(6)**,** 1418-1434. doi: 10.1287/orsc.1100.0594.

Shou, Y., Shan, S., Chen, A., Cheng, Y., and Boer, H. (2020). Aspirations and environmental performance feedback: a behavioral perspective for green supply chain management. *International Journal of Operations & Production Management* 40(6)**,** 729-751. doi: 10.1108/ijopm-11-2019-0756.

Singh, S., and Guha, M. (2018). Experiential learning: analyzing success and failures in Indian telecom sector. *Benchmarking: An International Journal* 25(9)**,** 3702-3719. doi: 10.1108/bij-07-2017-0199.

Smulowitz, S.J., Rousseau, H.E., and Bromiley, P. (2020). The behavioral theory of the (community‐oriented) firm: The differing response of community‐oriented firms to performance relative to aspirations. *Strategic Management Journal* 41(6)**,** 1023-1053. doi: 10.1002/smj.3123.

Sobrepere i Profitós, X., Keil, T., and Kuusela, P. (2022). The Two Blades of the Scissors: Performance Feedback and Intrinsic Attributes in Organizational Risk Taking. *Administrative Science Quarterly* 67(4)**,** 1012-1048. doi: 10.1177/00018392221117996.

Souder, D., and Bromiley, P. (2012). Explaining temporal orientation: Evidence from the durability of firms' capital investments. *Strategic Management Journal* 33(5)**,** 550-569. doi: 10.1002/smj.970.

Su, Y., and Si, S. (2015). What motivates financial innovation across countries? The influences of performance aspiration and economic freedom. *Management International Review* 55(4)**,** 563-587. doi: 10.1007/s11575-014-0237-0.

Su, Y., and Su, T. (2017). Performance aspiration, industrial search and R&D investment among chinese firms. *Chinese Management Studies* 11(2)**,** 270-283. doi: 10.1108/cms-02-2017-0032.

Sun, Y., and Qiu, Z. (2022). Positive Performance Feedback and Innovation Search: New Ideas for Sustainable Business Development. *Sustainability* 14(4). doi: 10.3390/su14042086.

Tarakci, M., Ateş, N.Y., Floyd, S.W., Ahn, Y., and Wooldridge, B. (2018). Performance feedback and middle managers’ divergent strategic behavior: The roles of social comparisons and organizational identification. *Strategic Management Journal* 39(4)**,** 1139-1162. doi: 10.1002/smj.2745.

Titus, V., Parker, O., and Covin, J. (2019). Organizational Aspirations and External Venturing: The Contingency of Entrepreneurial Orientation. *Entrepreneurship Theory and Practice* 44(4)**,** 645-670. doi: 10.1177/1042258719838473.

Tuggle, C.S., Sirmon, D.G., Reutzel, C.R., and Bierman, L. (2010). Commanding board of director attention: Investigating how organizational performance and CEO duality affect board members' attention to monitoring. *Strategic Management Journal* 31(9)**,** 946-968. doi: 10.1002/smj.847.

Tyler, B.B., and Caner, T. (2016). New product introductions below aspirations, slack and R&D alliances: A behavioral perspective. *Strategic Management Journal* 37(5)**,** 896-910. doi: 10.1002/smj.2367.

Vidal, E., and Mitchell, W. (2015). Adding by subtracting: The relationship between performance feedback and resource reconfiguration through divestitures. *Organization Science* 26(4)**,** 1101-1118. doi: 10.1287/orsc.2015.0981.

Villagrasa, J., Buyl, T., and Escribá-Esteve, A. (2018). CEO satisfaction and intended strategic changes: The moderating role of performance cues. *Long Range Planning* 51(6)**,** 894-910. doi: 10.1016/j.lrp.2017.12.002.

Wan, L., Li, R., and Chen, Y. (2021). Negative performance feedback and corporate venture capital: The moderating effect of CEO overconfidence. *Applied Economics* 54(16)**,** 1829-1843. doi: 10.1080/00036846.2021.1982133.

Wang, K., and Zhang, X. (2021). The effect of media coverage on disciplining firms’ pollution behaviors: Evidence from Chinese heavy polluting listed companies. *Journal of Cleaner Production* 280. doi: 10.1016/j.jclepro.2020.123035.

Wangrow, D.B., Kolev, K., and Hughes-Morgan, M. (2019). Not all responses are the same: How CEO cognitions impact strategy when performance falls below aspirations. *Journal of General Management* 44(2)**,** 73-86.

Wennberg, K., Delmar, F., and McKelvie, A. (2016). Variable risk preferences in new firm growth and survival. *Journal of Business Venturing* 31(4)**,** 408-427.

Wennberg, K., and Holmquist, C. (2008). Problemistic search and international entrepreneurship. *European Management Journal* 26(6)**,** 441-454. doi: 10.1016/j.emj.2008.09.007.

Xie, E., Huang, Y., Stevens, C.E., and Lebedev, S. (2019). Performance feedback and outward foreign direct investment by emerging economy firms. *Journal of World Business* 54(6). doi: 10.1016/j.jwb.2019.101014.

Xu, Y., and Zeng, G. (2020). Corporate social performance aspiration and its effects. *Asia Pacific Journal of Management*. doi: 10.1007/s10490-020-09706-0.

Xue, S., Zhang, L., Chen, H., and Yin, J. (2022). Does environmental underperformance duration affect firms' green innovation? Evidence from China. *Business Ethics, the Environment & Responsibility* 31(3)**,** 662-681. doi: 10.1111/beer.12434.

Yang, Z., Zhang, H., and Xie, E. (2017). Performance feedback and supplier selection: A perspective from the behavioral theory of the firm. *Industrial Marketing Management* 63**,** 105-115. doi: 10.1016/j.indmarman.2016.12.003.

Ye, Y., Yu, W., and Nason, R.S. (2021). Performance feedback persistence: comparative effects of historical versus peer performance feedback on innovative search. *Journal of Management* 47(4)**,** 1053-1081. doi: 10.4119727/01643920063921096126225.

Yu, W., Minniti, M., and Nason, R. (2018). Underperformance duration and innovative search: Evidence from the high‐tech manufacturing industry. *Strategic Management Journal* 40(5)**,** 836-861. doi: 10.1002/smj.2988.

Zhang, C.M., and Greve, H.R. (2018). Delayed adoption of rules: A relational theory of firm exposure and state cooptation. *Journal of Management* 44(8)**,** 3336-3363. doi: 10.4119727/016439210636176637737119.

Zhang, L. (2018). Risk or uncertainty: The effect of performance on risk taking in the mutual fund industry. *Working Paper*.

Zhong, X., Ren, L., and Song, T. (2021). Beyond Market Strategies: How Multiple Decision-Maker Groups Jointly Influence Underperforming Firms’ Corporate Social (Ir)responsibility. *Journal of Business Ethics*. doi: 10.1007/s10551-021-04796-2.

Zhong, X., Ren, L., and Song, T. (2022). To cheat when continuously missing aspirations: Does CEO experience matter? *Asia Pacific Journal of Management*. doi: 10.1007/s10490-022-09805-0.

**
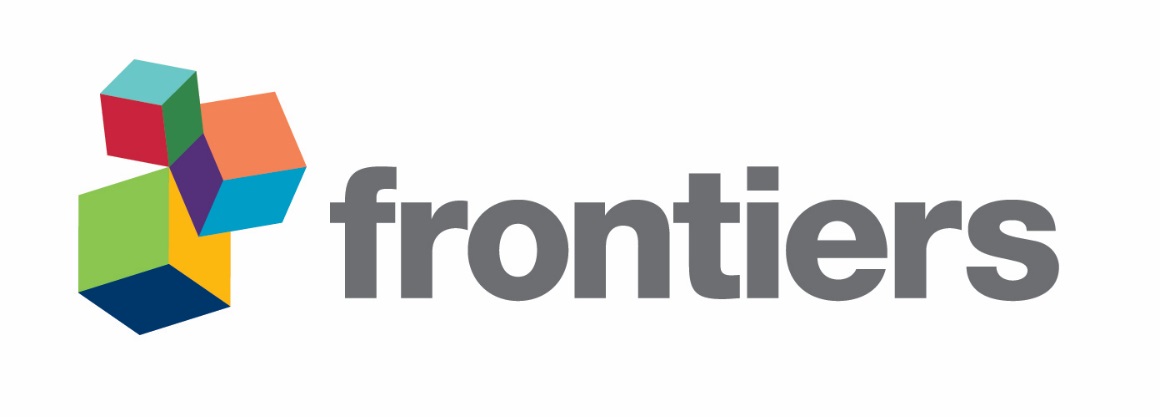
**
